# Phylogenetic and morphological relationships between nonvolant small mammals reveal assembly processes at different spatial scales

**DOI:** 10.1002/ece3.1407

**Published:** 2015-01-25

**Authors:** André Luís Luza, Gislene Lopes Gonçalves, Sandra Maria Hartz

**Affiliations:** 1 Programa de Pós Graduação em Ecologia, Universidade Federal do Rio Grande do SulAv. Bento Gonçalves 9500, CEP: 91501-970 - Post-Office Box: 15007, Porto Alegre, Rio Grande do Sul, Brazil; 2 Departamento de Genética, Universidade Federal do Rio Grande do SulAv. Bento Gonçalves 9500, CEP: 91501-970, Porto Alegre, Rio Grande do Sul, Brazil; 3 Instituto de Alta Investigación, Universidad de TarapacáAntofagasta, 1520, Arica, Chile

**Keywords:** Coexistence, Didelphimorphia, environmental filters, niche overlap, Rodentia, similarity limitation

## Abstract

The relative roles of historical processes, environmental filtering, and ecological interactions in the organization of species assemblages vary depending on the spatial scale. We evaluated the phylogenetic and morphological relationships between species and individuals (i.e., inter- and intraspecific variability) of Neotropical nonvolant small mammals coexisting in grassland-forest ecotones, in landscapes and in regions, that is, three different scales. We used a phylogenetic tree to infer evolutionary relationships, and morphological traits as indicators of performance and niche similarities between species and individuals. Subsequently, we applied phylogenetic and morphologic indexes of diversity and distance between species to evaluate small mammal assemblage structures on the three scales. The results indicated a repulsion pattern near forest edges, showing that phylogenetically similar species coexisted less often than expected by chance. The strategies for niche differentiation might explain the phylogenetic repulsion observed at the edge. Phylogenetic and morphological clustering in the grassland and at the forest interior indicated the coexistence of closely related and ecologically similar species and individuals. Coexistence patterns were similar whether species-trait values or individual values were used. At the landscape and regional scales, assemblages showed a predominant pattern of phylogenetic and morphological clustering. Environmental filters influenced the coexistence patterns at three scales, showing the importance of phylogenetically conserved ecological tolerances in enabling taxa co-occurrence. Evidence of phylogenetic repulsion in one region indicated that other processes beyond environmental filtering are important for community assembly at broad scales. Finally, ecological interactions and environmental filtering seemed important at the local scale, while environmental filtering and historical colonization seemed important for community assembly at broader scales.

## Introduction

Animal taxa and their adaptations to environmental conditions are dependent on the evolutionary history of ancestral lineages, so that related species share many morphological characters and ecological niche requirements (Webb et al. 2002). This pattern of niche conservatism imposes restrictions on the occupancy patterns of current lineages in certain habitats or generates strong competition due to niche overlap among phylogenetically close taxa (Cavender-Bares et al. 2009; Davies and Buckley 2011). Patterns of coexistence based on functional and phylogenetic similarities may reflect different structuring forces, which are generally attributed to deterministic processes such as environmental filtering and ecological interactions (Diamond 1975; Keddy 1992; Webb et al. 2002), or stochastic forces that are commonly independent of functional and phylogenetic affinities among taxa (Hubbell 2006).

Local-scale variations in functional and phylogenetic structures of communities allow recognition of factors leading to competitive exclusion or to the coexistence of ecologically similar taxa (Lovette and Hochachka 2006). Environmental filters operating on conserved traits during the evolutionary history of lineages result in clustered communities, where organisms coexisting locally are more functionally and phylogenetically related than would be expected if the communities were randomly assembled (Webb et al. 2002; Kraft et al. 2007). Environmental filtering operating on convergent traits generates functional clustering but phylogenetic repulsion, which means that nonrelated taxa share traits enabling them to coexist in communities that are affected by severe environmental factors (Keddy 1992; Webb et al. 2002; Pavoine and Bonsall 2011). Ecological interactions acting on functional traits conserved through phylogeny tend to generate functional and phylogenetic repulsion, reflecting a process of similarity limitation in species co-occurrence (Diamond 1975; Kelt et al. 1995; Webb et al. 2002). However, similarity limitation may generate phylogenetic clustering when the strongest competitive abilities are phylogenetically conserved and when competitive exclusion of the weaker species occurs (Mayfield and Levine 2010). Although ecological interactions acting on traits that vary widely across the phylogeny tend to result in character displacement among sympatric species (Davies et al. 2012), the resulting patterns make it difficult to predict any assembly processes (Webb et al. 2002). Finally, if co-occurrence patterns at local scale do not differ from those randomly expected, the irrelevance of functional similarities and phylogenetic relatedness, that is, ecological equivalence on community assembly process is implied (Webb et al. 2002; Hubbell 2006). Because the importance of community assembly processes varies in a scale-dependent manner, it is interesting to define how the coexistence patterns behave at various scales (Gomez et al. 2010). Evaluation of broad-scale variations in community structures may help to define historical, ecological, and biogeographic processes constraining the regional species pool (Lovette and Hochachka 2006; Cavender-Bares et al. 2009).

Grassland-forest ecotones are components of vegetation mosaics where forest and grassland physiognomies are in contact (Luza et al. 2014). Ecotones may originate from a forest expansion process (Oliveira and Pillar 2004), which allows the establishment of woody plants (nurse plants) and begins the formation of forest patches on grasslands (Duarte et al. 2006). Woody nucleation is observed worldwide in regions with high rainfall levels (Bond and Parr 2010). As forest expansion characterizes a regional dynamic, phylogenetic and functional structures of animal assemblages occurring in regions containing grassland-forest ecotones can help to define the importance of environmental restrictions imposed by ecological filters and/or historical processes linked to colonization of lineages composing the regional pool. In order to infer processes structuring assemblages along a spatial gradient, we performed a nested sampling by distributing sampling units of nonvolant small mammals over three regions with grassland-forest ecotones in southern Brazil.

Species of Rodentia and Didelphimorphia frequently co-occur in small mammal assemblages and may interact because of their many ecological affinities (Cooper et al. 2008). Additionally, many congeners occur locally (e.g., species of *Akodon*), making them an appropriate group to explore the mechanisms allowing the coexistence of morphologically and phylogenetically related taxa along environmental gradients. Previous studies have demonstrated that species of small mammals in highly heterogeneous environments tend to coexist more frequently than would be expected from a random distribution, while the inverse pattern is observed in less heterogeneous environments (Stevens et al. 2012). Evidence indicates a common trend toward phylogenetic repulsion in mammal assemblages, due to competitive interactions that prevent related species from coexisting (Gotelli and McCabe 2002; Cooper et al. 2008). However, few studies have evaluated mammal coexistence at fine scales (e.g., Stevens et al. 2012) and combined functional and phylogenetic aspects (Fritz and Purvis 2010; Gomez et al. 2010; Cooper et al. 2011), as not all functional traits are phylogenetically conserved (Losos 2008; Gomez et al. 2010). Furthermore, trait values among and within species tend to vary along environmental gradients such as ecotones, enabling us to assess assembly processes using species- and individual-based approaches, due to the action of contrasting selective forces which may result in differences in ecological requirements between species and individuals (Violle et al. 2012). Individual variability may indicate phenotypic and evolutionary trends tracked by species populations according to differences in environmental conditions across gradients (Arnold 1983). Thus, the study of grassland-forest ecotones may reveal underlying processes that influence patterns of coexistence in small mammal assemblages, as evaluated through interspecific and intraspecific approaches at several scales.

Our study aimed to assess the assembly processes underlying the coexistence of nonvolant small mammal at different scales. Using phylogenetic relationships among species and morphological similarities among species and individuals, we asked whether small mammal assemblages are deterministically structured at local, landscape, and regional scales according to variation of morphological and phylogenetic relationships. We observed variations in species composition, species richness, and total abundance in nonvolant small mammal assemblages across grassland-forest ecotones at different spatial scales, which were related to changes in vegetation heterogeneity and structure and in litter depth, which in turn are affected by ecological disturbances (A. L. Luza, unpubl. data). Habitat structure and regional processes related to forest expansion might result in distinct coexistence patterns, revealed through phylogenetic and morphological relationships.

Grasslands are vertically less heterogeneous than edge and forest habitats and have higher vegetation density and herbaceous horizontal heterogeneity than edge and forest, mainly in grassland that has not undergone disturbances (e.g., burning and intensive cattle grazing). Grasslands are also more susceptible to the effects of seasonality or disturbances affecting habitat structure, because highly inflammable senescent biomass accumulates during the growing season, and fires generally die out on the forest edge (Pillar and Quadros 1997). Thus, such environmental filtering might select organisms with small body length and long tails and feet (e.g., *Oligoryzomys nigripes*), which are highly mobile and tolerant to harsh filters related to strong predation risk, and are efficient in searching for resources in habitats with low vegetation cover (Taraborelli et al. 2003; Pedó et al. 2010). Thus, we expected phylogenetic and morphological clustering in grasslands. Conversely, in forest and edge habitats, we expected a pattern of low ecological similarity (phylogenetic and functional repulsion) due to effective niche partition, high vertical heterogeneity, and the absence of severe environmental filters (Gotelli and McCabe 2002; Graham et al. 2009). Thus, under different spatial scales, herein we investigated whether assemblages are phylogenetically and functionally clustered at broad scales.

## Material and Methods

### Study area

**Figure 1:**
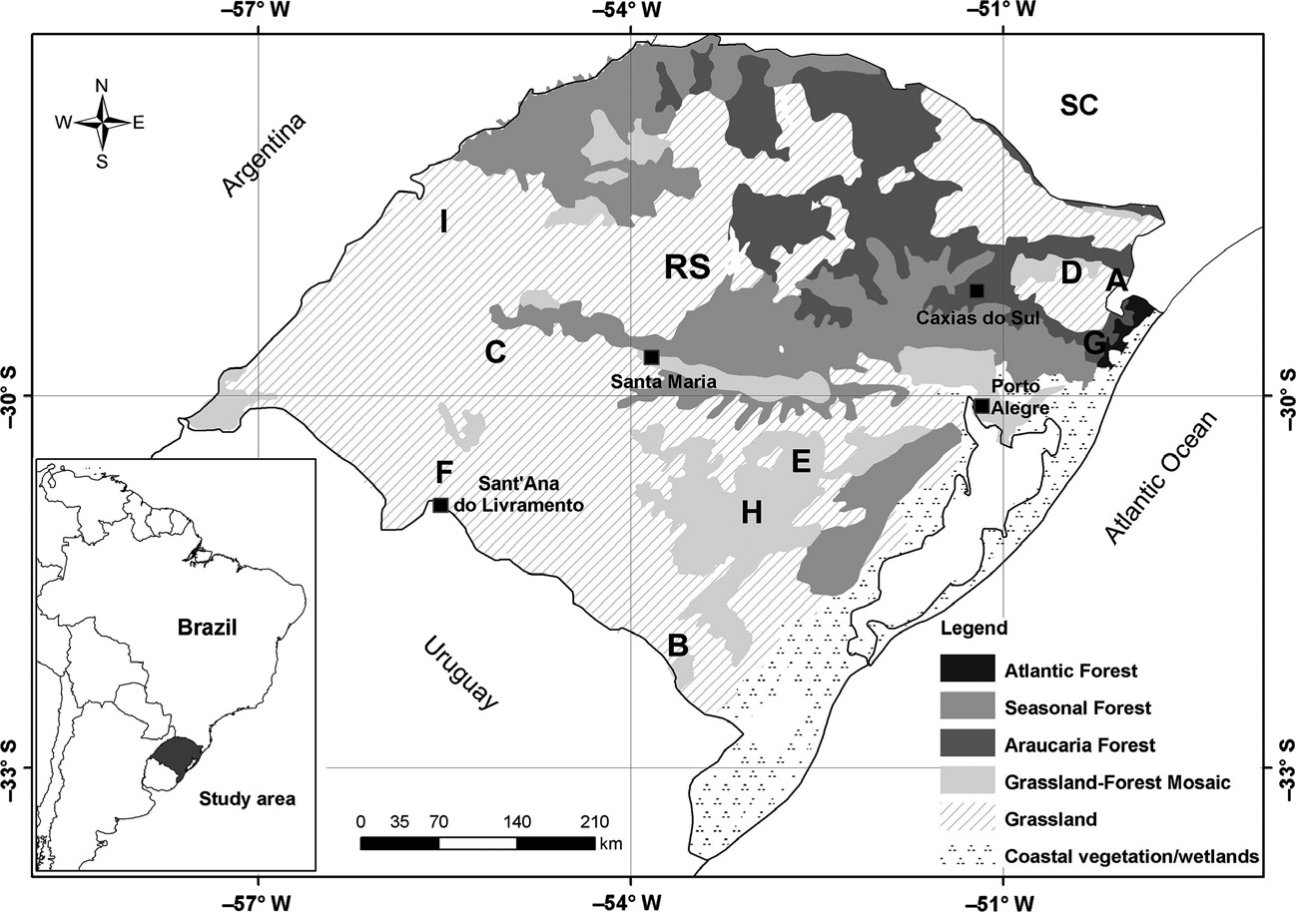
Map of the dominant vegetation physiognomies of southern Brazil (according to IBGE, 2004). Letters A–I indicate landscapes and represent the order of the temporal sequence of small mammal sampling. Atlantic Forest biome (region A: landscapes A, D, and G) and Pampa biome (Serra do Sudeste: region B: landscapes B, E, and H; and Campanha: region C: landscapes C, F, and I).

The study was conducted along grassland-forest ecotones in nine localities within three regions in southern Brazil (Fig.1). The sites have been affected by livestock grazing and trampling and/or by fire. Grassland-forest ecotones are a common landscape feature in the Campos Sulinos (= southern Brazilian grasslands) physiognomy, which occurs in the Pampas and Atlantic Forest biomes (Boldrini 2009). Ecotones between Brazilian Upland Grasslands and Araucaria Forest occur in the Atlantic Forest biome, while several physiognomies of the Brazilian Northern Campos (included in the Río de La Plata Grasslands) comprise ecotones with deciduous forest in the Serra do Sudeste and Campanha regions, both in the Pampas biome (Boldrini 2009). The climate across this region is predominantly mild mesothermal temperate (mean annual temperature between 10 and 15°C) and mean mesothermal (mean <10°C) at high altitudes (1000 m a.s.l.) (Nimer 1979; IBGE 2002).

### Sampling of nonvolant small mammals

In each of the nine localities (the landscape scale), two 140 × 140 m grids were established, at least 1 km apart from each other. Sampling of nonvolant small mammals took place in three campaigns: (1) October 2011 and January and February 2012 (Fig.1 – # A, B, and C), (2) March through April 2012 (Fig.1 –# D, E, F), and (3) September and December 2012 and February 2013 (Fig.1 – # G, H, I – Fig.1), avoiding nights with full moon phases due to light conditions increased predation risk (Griffin et al. 2005). Each locality was sampled in one of these periods. In each campaign, localities from all three regions were sampled (Fig.1), thus controlling for possible temporal effects on species composition due to seasons or years among regions (see further details in *Data Analysis*). Our basic sampling units were 140-m transects parallel to the forest edge. On each transect, eight capture points 20 m apart were established (Fig. S1). Each capture point was composed of one Sherman (25 × 8 × 9 cm) and one Tomahawk trap (45 × 17.5 × 15 cm) installed on the ground, 0.5 m apart and with trap openings facing in opposite directions. A mixture of bananas, peanuts, sardines, cod-liver oil, vanilla essence, and corn meal was used as bait. Eight transects were arranged across the interface between grassland and forest, forming a 140 × 140 m grid (Fig. S1). Small mammals were sampled during 5 days in each grid. Traps were checked in the morning and in the afternoon, totaling 1280 traps/grid. Trapped animals were identified, marked, and released at the same point. We used a modification of the *Mossa australiana* system (Mangini and Nicola 2003) to mark and recapture the small mammals. The animals captured in the grassland transects were marked with small cuts on the outer part of the left ear, while forest animals were marked on the right ear. This method allowed us to define which species belonged to the grassland and which to the forest assemblages, as well as which ones were present in both habitats. Because of the coexistence of cryptic species (mainly members of *Akodon*) and the scarcity of grassland-forest ecotone studies in southern Brazil, DNA analysis was used to confirm the field identifications (based on external morphology). Sequences of the mitochondrial cytochrome *b* gene were generated from captured individuals and blasted in GenBank and in the local database of the Cytogenetic and Evolution Laboratory of the Universidade Federal do Rio Grande do Sul. Samples were deposited at the Molecular Ecology Laboratory of the Universidade Federal do Rio Grande do Sul, and one sequence per species was deposited in GenBank (accession numbers KJ936941–KJ936960).

### Phylogenetic tree

**Figure 2:**
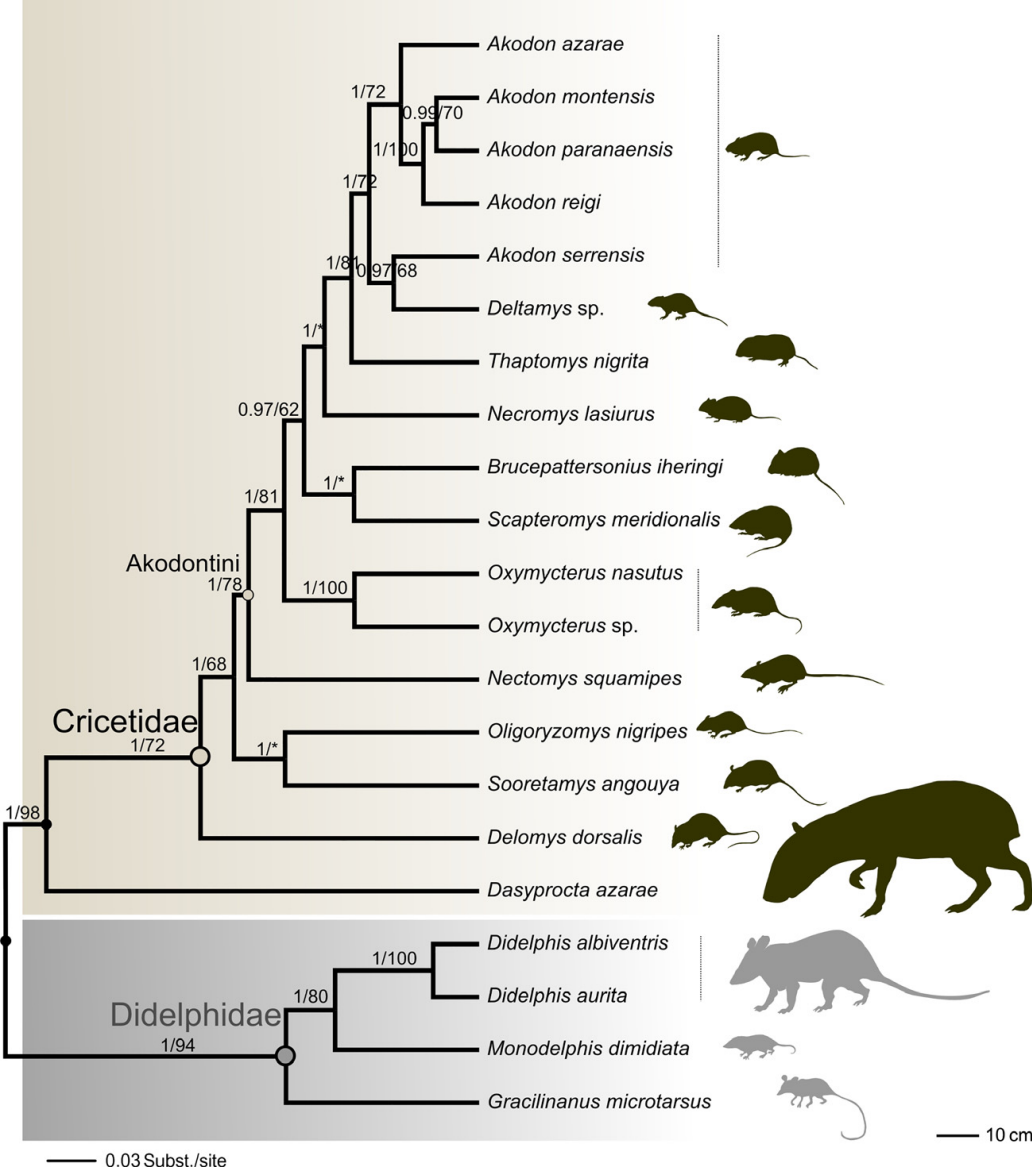
Consensus Bayesian tree showing phylogenetic relatedness between 21 small mammal species from grassland-forest ecotones. A total of 3.1 Kb from two mitochondrial genes (cytochrome oxidase I [COI], cytochrome *b* [Cyt-*b*]) and one nuclear (interphotoreceptor retinoid-binding protein [IRBP]) locus were used to reconstruct the tree. Values above branches indicate Bayesian posterior probabilities/bootstrap obtained from maximum likelihood. Animal shadows on the left are draw on scale to represent variation on body size.

In order to evaluate the evolutionary relationships among small mammal species and infer community assembly processes across several scales, we reconstructed a molecular phylogeny using 3.1 kb of multilocus DNA sequences, mostly using data previously available for specimens in public databases (e.g., GenBank). We generated our own data for three species endemic to the study area: *S. meridionalis*, recently described (Quintela et al. 2014) and two undescribed taxa, *Deltamys* sp. and *Oxymycterus* sp. A total of three genes, two mitochondrial (cytochrome *b* [Cyt-*b*], 1140 bp) and (cytochrome oxidase I [COI], ca. 700 bp) and one nuclear (interphotoreceptor retinoid-binding protein [IRBP], ca. 1200 bp), were concatenated from specimens obtained from GenBank, including 18 of the 21 taxa surveyed in this study (Appendix S1). Phylogenetic trees were reconstructed using Bayesian inference (BI) through BEAST 2.01 (Bouckaert et al. 2014) (Fig.2). Concatenated loci were unlinked to allow for variation in substitution models, and the clock models for the mtDNA were linked to account for their presumed single hierarchical history. The branch lengths were allowed to vary under a relaxed clock model with an uncorrelated lognormal distribution (Drummond et al. 2006). The analysis was run using a Yule species tree prior and the GTR model of nucleotide substitution [according to the Akaike information criterion run on the program jModeltest (Posada 2008)], with four rate categories. The Markov chain Monte Carlo (MCMC) was run for 50 million generations and repeated 10 times to test for chain convergence, and priors exceeded 200 to ensure effective sample sizes (ESS). Burn-in was determined in Tracer 1.5 (Drummond and Rambaut 2007) based on ESS and parameter trajectories and was then removed in TreeAnnotator. The consensus tree was observed and edited in FigTree 1.4 (Rambaut 2009). Nodes with Bayesian credibility values (BC) ≥95% were considered strongly supported. Phylogenetic trees were also reconstructed using maximum likelihood (ML) in the software PHYML 3.0 (Guindon et al. 2010) using the GTR model of sequence evolution. Monophyly confidence limits were assessed with the bootstrap method (Felsenstein 1985) at 60% cutoff after 1000 bootstrap iterations. Major relationships in the consensus tree between the two groups of small mammals were recovered according to Fabre et al. (2012). Most importantly, internal relationships at genus and species level within each subgroup reflect relationships as previously proposed (e.g., D'Elia 2003; Weksler 2003; Jansa and Weksler 2004; Steppan et al. 2004; Leite et al. 2014; Mitchell et al. 2014).

### Morphological traits

In order to assess competitive interactions and similarities in ecological tolerances among species and individuals based on morphological traits, we used two approaches. The first was the species-based functional approach (Petchey and Gaston 2002), in which a trait value for trapped species (21 species) consists of the mean for all adult individuals measured in the sampling period. For species recorded only once, we used the individual trait value. The second was the individual-based approach (Cianciaruso et al. 2009), in which we used the trait values of each individual captured (236 individuals). The latter assumes that a trait condition of a given individual in a particular gradient portion can differ from another individual belonging to the same species but situated in another part of the gradient (Violle et al. 2012), providing distinct or complementary information on individual performance and assembly processes shown by the species mean approach (Cianciaruso et al. 2009).

We used morphological attributes for 21 species, because such attributes are easily measurable and in some situations are related to organism performance (Arnold 1983). Traits used to represent morphology-based coexistence patterns between species and individuals were related to habitat utilization and locomotion. Cursorial species/individuals generally have long bodies (Christoff et al. 2009), because longer bodies may restrict locomotion in some shrub and tree substrates (Shapiro et al. 2014). Scansorial species tend to have a long tail (prehensile in some marsupials) for balance while moving along tree branches (Szalay 1994) and as an swimming accessory for semiaquatic species (e.g., *Nectomys squamipes*) (Christoff et al. 2009). The hind foot length was used to represent the modes of locomotion. A large hind foot characterizes jumpers, while creepers or walkers have a small foot (Taraborelli et al. 2003; Samuels and Van Valkenburgh 2008). A long foot can also indicate climbing ability (Shapiro et al. 2014). Claw size was used to characterize diggers or the individual capacity for grasping tree branches (Szalay 1994; Samuels and Van Valkenburgh 2008). Ear size (auricle) was used to represent the semifossorial habit (surface-foraging burrowers), which includes mainly species with small ears (Szalay 1994; Lange et al. 2004). All five traits were measured in millimeters (Table S1). To reduce errors, all measurements were made by the same person (ALL). In order to remove the influence of discrepancies in organism body size (see Fig.2) on morphological traits, we divided each trait value by the cubic root of the individual body mass or species mean body mass, depending on the approach (Samuels and Van Valkenburgh 2008). Allometry was evaluated using slope values (*b*) from linear regressions between trait values against cubic root of body mass. Negative allometry was identified in all species and individual traits (*b* < 1), although species body and foot length (Fig. S2) and individual claw size (Fig. S3) approached isometric scaling with body mass (*b* ≈ 1). Additionally, traits were standardized by mean and standard deviation, and the UPGMA clustering based on Euclidian distance was used to build a dendrogram of species and individuals by their traits for subsequent analysis. The phylogenetic signal tested through Blomberg et al.'s (2003) showed that close relatives are more similar in posterior foot length and claw size than expected under Brownian motion evolution (*K* = 1.18 and *K* = 1.4, respectively; *P* = 0.002). Close relatives were less similar in tail and body length than expected (*K* = 0.85; *P* = 0.001; *K* = 0.62; *P* = 0.035, respectively), while ear size showed no phylogenetic pattern (*K* = 0.35; *P* = 0.73). As traits exhibited different levels of phylogenetic signal, indexes of morphological diversity were calculated using two levels: all traits together and each trait separately.

### Data analysis

Our composition matrix includes presence/absence data for 21 species/236 individuals across transects. In addition to species/individual presence recorded on a given transect on the first day of sampling, we further considered the presence of species/individuals moving between transects recorded through successive recaptures on successive days of sampling at each site. Thus, an individual or species may be recorded on a different transect from where it was first trapped. The small mammal sampling was spatially distributed, and each grid was sampled only once, which could introduce a temporal effect in the species detection. To compensate for this effect and correctly compare species composition among regions, we performed a fieldwork rotation, and then included the rotation sequence as a block factor (Fig.1 – block 1: # A, D, and G; block 2: # B, E, and H; and block 3: # C, F, and I) in a MANOVA with restricted permutations (Pillar and Orloci 1996). Permutations were restricted within blocks composed by the six grids surveyed in each campaign. The MANOVA was based on the Jaccard similarity matrix between grids. The resulting MANOVA showed that region significantly explained the compositional similarity between sampling grids before and after removing the influence of sampling period (Region SS = 12.74; *P* = 0.0001), because the temporal influence was small (Block SS = 3.26; *P* = 0.0001).

We tested the hypothesis that nonvolant small mammal assemblages were deterministically structured by generating random index values, taking into account the phylogenetic relationships and the trait similarities. The phylogenetic and functional patterns on different scales were assessed using the approach proposed by Pavoine & Bonsall (Pavoine and Bonsall 2011). As phylogenetic and functional measurements are based on dendrograms and trees or on distance matrices, Pavoine and Bonsall reasoned that it is permissible to use the same indexes to describe both the phylogenetic and functional structure of communities. To measure the phylogenetic and morphological distances between coexisting taxa, we used the Phylogenetic (PD) (Faith 1992) and Functional Diversity (FD) (Petchey and Gaston 2002) indexes and the Mean Pairwise Phylogenetic Distance (MPD), the Functional Distance (MFD), and the Mean Nearest Pairwise Phylogenetic and Functional Distance indexes between taxa co-occurring in the communities (MNTD) (Webb et al. 2002). PD and FD sum the dendrogram or tree branch lengths linking taxa coexisting in a assemblage, compounding a morphological and phylogenetic richness index (Petchey and Gaston 2002). MPD and MFD express, respectively, mean relatedness and mean morphological distance between co-occurring taxa, revealing patterns that occur throughout a phylogenetic tree or morphological dendrogram (Kraft et al. 2007). The MNTD measures the mean phylogenetic and morphological distance between neighbor taxa or congeners and is more efficient in detecting patterns related to similarity limitation (Kraft et al. 2007). Indexes were computed with the “ses.pd”, “ses.mpd”, and “ses.mntd” functions implemented in the Picante package (Kembel et al. 2010) in the R 2.12.2 environment (R Development Core Team 2011). Indexes of mean pairwise distances for species and individuals – MPD, MFD, and phylogenetic/morphological – MNTD – were multiplied by -1, equaling the NRI (Net relatedness index) and NTI (Nearest taxon index) indexes (Webb et al. 2002). These index values were used to measure the standardized effect size (SES) of the mean and standard deviation expected for random communities in the observed phylogenetic and morphological distances. The null hypothesis predicts that the mean SES is equal to zero, which characterizes communities that are not deterministically structured (Gotelli and McCabe 2002). The SES distribution varies from 1 to −1, and positive values indicate clustering, while negative values indicate phylogenetic and morphological repulsion, thus providing evidence for the ecological processes such as environmental filters or biotic interactions influencing the observed patterns (Webb et al. 2002; Pavoine and Bonsall 2011). Random phylogenetic and morphological indexes were generated through 1000 permutations of the independent swap algorithm (Gotelli 2000).

In order to evaluate the coexistence patterns across several scales, we first calculated phylogenetic species-based indexes (PD, NRI, and NTI) and species- (FD, NRI, and NTI) and individual-based morphological diversity indexes (iFD, iNRI, and iNTI) for each transect where more than two individuals or species were captured. We then evaluated community structure across three scales: local (transects across the grassland-forest ecotone), landscape (transects inside the two sampling grids within the same landscape), and regional (transects inside the six grids within the same region). To scale-up coexistence patterns observed for each transect, we grouped all transects replicated on the 15 grids with captures located in the same portion of the gradient, composing the local scale (transects 1–8, from the grassland interior to the forest interior; Fig. S1), and calculated a mean index value and the 95% confidence interval values (CI). By doing so, we were able to vary the species or individual occurrences among the grouped sampling units, while keeping unaltered the phylogenetic and morphological pools of species. At the landscape scale, we grouped each sampling transect located in the two grids in the same landscape, and at the regional scale, we combined all index values belonging to the six grids situated in each of the three regions. For the landscape and regional scales, we also calculated the mean index values and the associated 95% CI. MANOVA was performed in the software Multiv 2.95 (available at http://ecoqua.ecologia.ufrgs.br/ecoqua/MULTIV.html). All other analyses were performed in the R 2.12.2 environment (R Development Core Team 2011).

## Results

A sampling effort of 19,877 trap-nights resulted in 306 captures of 236 individuals and a mean capture success of 1.3%. Seventeen species belonged to the order Rodentia and four to Didelphimorphia (Table S2). The most frequent species at the sites were *Oligoryzomys nigripes* (Olfers 1818), *Didelphis albiventris* (Lund 1840), *Akodon montensis* (Thomas 1913), and *Oxymycterus nasutus* (Waterhouse 1837) (Table S2). Transects showing the highest mean species richness (mSR) were those situated in grassland (transect 2; mSR = 1.6 ± 1.45) and forest interior (transect 8; mSR = 1.4 ± 1.55), while the lowest mSRs was recorded near the forest edge (transect 3; mSR = 1.13 ± 1.68) and forest interior (transect 6; mSR = 1.2 ± 1.01). At the landscape scale, ecotones between Upland Grassland and Araucaria Forest (area G) showed an mSR of 10.5 ± 0.7, and the mSR in another Araucaria Forest area (D) was 7 ± 1.4. No species were captured in area F, and only 1.5 ± 0.7 species were recorded in area I. Regionally we recorded a mean of 7.5 ± 2.6 species in the Araucaria Forest region, 2.3 ± 1.21 in Serra do Sudeste and 1.16 ± 1.6 in Campanha. The two grids of landscape F and grid 1 of area C, both in the Campanha region, were not included in the analysis because no individuals were collected (Fig.1, Table S2). Because the pairwise distance is necessary to calculate these indexes, and some species or individuals did not co-occur on some transects, it was not possible to calculate CIs for landscapes B and C using the individual approach, and landscapes B, C, and I using the species approach.

**Figure 3:**
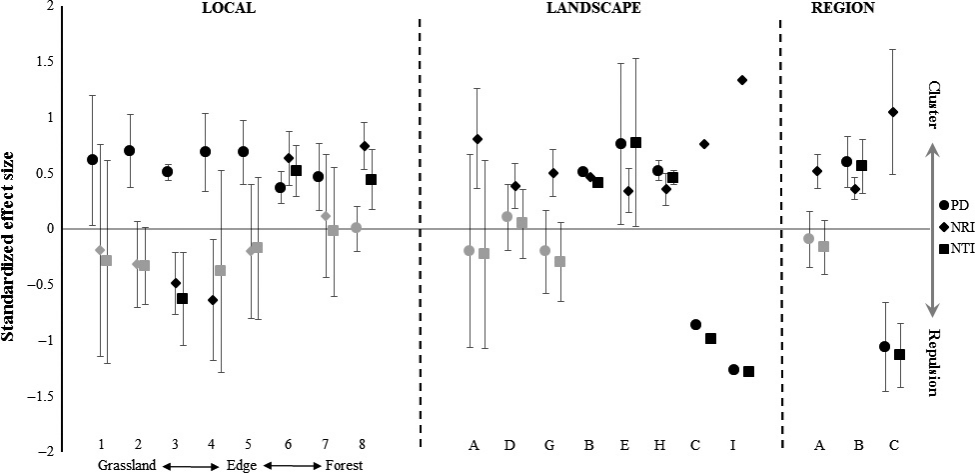
Species-based phylogenetic approach showing the structure of nonvolant small mammal assemblages at three scales. Bars are equivalent to confidence intervals of 95%. Black symbols indicate index values different from the random expectation. PD = Phylogenetic Diversity; NRI = Net relatedness index; NTI = Nearest taxon index.

Nonvolant small mammal assemblages were structured according to phylogenetic and morphological similarities across the spatial scales evaluated. The results at local scale showed a pattern of phylogenetic clustering in the grasslands and forest, and a repulsion pattern near the forest edge (transects 3 and 4) (Fig.3). The sum of phylogeny branch lengths connecting all species coexisting in each assemblage (PD) indicated a clustering structure for almost the entire gradient (transects 1–7; Fig.3), showing that phylogenetically related species coexisted more than would be expected by chance. Mean phylogenetic distance between species (NRI) and phylogenetic distance between nearest taxa (NTI) phylogenetic indexes were higher than randomly expected only for transects 6 and 8, and lower than randomly expected for transect 3, while the mean NRI was also lower for transect 4 (Fig.3). These findings suggest the existence of phylogenetic repulsion in grassland transects situated near the forest edge, where phylogenetically related species coexist less than expected by chance. At the landscape scale, we observed predominantly phylogenetic clustering (Fig.3). The mean values of PD showed phylogenetic clustering in all landscapes except for those in the Campanha region (C and I); mean NTI was lower than randomly expected in areas C and I, while mean NRI was higher in these areas. At regional scales, the Phylogenetic Diversity and Near Taxon Index showed clustering in Serra do Sudeste and repulsion in Campanha. Mean Net Relatedness Index indicated clustering in three regions (Fig.3).

**Figure 4:**
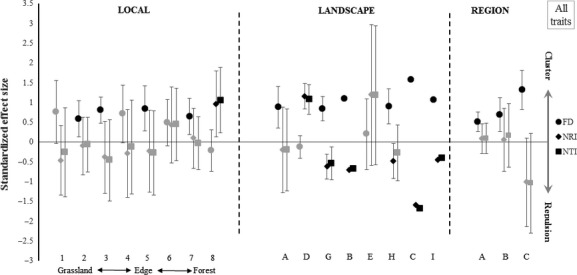
Species-based morphologic approach showing the structure of nonvolant small mammal assemblages at three scales. Bars are equivalent to confidence intervals of 95%. Black symbols indicate index values different from the random expectation. FD = Species Functional Diversity; NRI = Net relatedness index; NTI = Nearest taxon index.

Coexistence patterns based on individual and species morphological traits showed subtle differences from those observed by the phylogenetic approach. Intraspecific and interspecific scaling of traits with body mass revealed negative allometry, indicating that traits grow at lower rates than body growth (Figs. S2, S3). Patterns of coexistence using each trait separately were very similar to patterns revealed by analysis using all traits, independently of whether a given trait showed no phylogenetic signal (ear size) and was either more (foot length and claw size) or less conserved than expected from the Brownian motion model (tail and body length). The results of analyses using all traits are shown. Patterns based on each trait separately can be found in the supplementary results (Appendix S2). The sum of dendrogram branch lengths connecting all species coexisting in each assemblage (FD) showed a predominant clustering of small mammal morphologies throughout the grassland-forest gradient (Fig.4). The mean morphological distance between species (NRI) and mean distance between mostly similar species (NTI) were higher than randomly expected only for the forest interior (transect 8). At the landscape scale, the mean Functional Diversity index (FD) was higher than expected by chance in six landscapes (Fig.4). The mean NRI and NTI were higher than expected by chance only in landscape D and were lower in one landscape in the Araucaria Forest region (G), one area in Serra do Sudeste (B), and two areas in Campanha (C and I), while the mean morphological distance (mean NRI) was lower than randomly expected in H. The morphological structure (mean FD) based on the species approach showed clustering in all regions, while NRI and NTI did not differ from random expectations (Fig.4). A repulsion pattern was evidenced through species ear size in Serra do Sudeste and species foot length in Campanha (Appendix S2).

**Figure 5:**
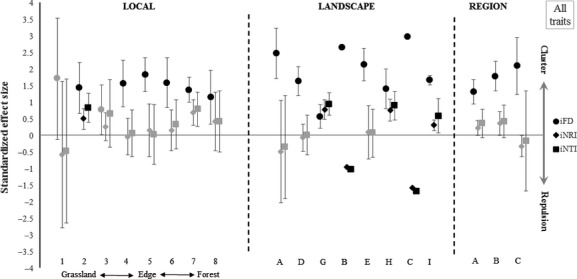
Individual-based morphologic approach showing the structure of nonvolant small mammal assemblages at three scales. Bars are equivalent to confidence intervals of 95%. Black symbols indicate index values different from the random expectation. iFD = Individuals Functional Diversity; iNRI = individual net relatedness index; iNTI = individual nearest taxon index.

The individual-based approach at the local scale allowed us to confirm that morphological clustering was the dominant pattern throughout the gradient (Fig.5). Patterns of coexistence between individuals employing each trait separately were similar to those exhibited by all traits. The results using all traits are shown. Patterns employing each trait separately can be assessed in the supplementary results (Appendix S2). We observed clustering of morphologies along the grassland-forest ecotone, as shown by the mean distance between individuals (iFD), indicating that morphologically similar individuals co-occurred more than expected by chance (Fig.5). The mean iNRI and iNTI were higher than randomly expected only in a grassland transect (2). Mean iFD values were higher than randomly expected in all landscapes, and mean values of iNRI were lower only in B and C (Fig.5). Finally, the mean iFD showed individual-based functional clustering in all regions, whereas iNRI and iNTI did not differ from random expectations (Fig.5). Individual claw size revealed a repulsion pattern in the Campanha region (Appendix S2).

## Discussion

The nonvolant small mammal assemblages were structured phylogenetically and morphologically at all scales evaluated. Our results showed negative allometry in morphological traits in both intraspecific and interspecific comparisons. Foot length and claw size were more phylogenetically conserved than expected by Brownian motion. However, the employment of each trait separately, owing to differences in phylogenetic signals, did not produce patterns much different from those generated using all morphological traits together. The index of morphological diversity for species (FD, NRI, and NTI) and individual traits (iFD, iNRI, and iNTI) revealed a predominant clustering of morphologies throughout the gradient, indicating that marsupial and rodent species coexisting in an ecotone assemblage are more morphologically similar than expected. The index of phylogenetic diversity (PD) revealed clustering along the grassland-forest gradient. The mean phylogenetic distance (NRI and NTI) showed phylogenetic repulsion in transects situated near the forest edge, and clustering on other grassland and forest transects. Clustering of morphologies recorded at three scales can be reinforced by the high frequency of occurrence of some marsupial (*Didelphis albiventris* and *Monodelphis dimidiata*) and rodent species (*Oligoryzomys nigripes*, *Oxymycterus nasutus,* and *Akodon* spp.) in ecotone assemblages, which share many morphological similarities after the influence of body size is removed. The high probability of coexistence among these species may explain the increased phylogenetic distance between the marsupial and rodent lineages co-occurring in the edge assemblages. The high incidence of co-occurrence of a few species might underlie the low variability in species composition, consequently increasing morphological similarities between species and individuals coexisting in small mammal assemblages. In some landscapes (mainly those from the Serra do Sudeste and Campanha regions), we had low trapping success. These regions suffer from recurrent rainfall deficits (Pillar and Quadros 1997), which may have resulted in a period of low population density of many species and cause problems in detection. This can result in infrequent species co-occurrence, likely affecting values of phylogenetic and morphological indexes as well as the associated confidence intervals. It also might explain the wide confidence intervals associated with all indexes and the frequent high values of PD and FD. Although NRI and NTI values in many cases did not differ from expectations, these indexes seemed more able to capture even subtle differences in morphological and phylogenetic distances between co-occurring taxa than PD and FD. Finally, another possible constraint on our results is related to the methods and indexes used, because they do not distinguish between biotic (weaker competitor exclusion) and abiotic processes (environmental filtering) producing the clustering pattern (Mayfield and Levine 2010).

The observed phylogenetic clustering is in agreement with our initial hypothesis of environmental filtering in grasslands. As initially supposed, we found low phylogenetic similarity between species co-occurring near the forest edge, but not in the forest interior. Differently from expectations, we found clustering of species and individual morphologies across the entire grassland-forest gradient, which might indicate low intraspecific variability in the morphological traits evaluated. Phylogenetically closer small mammal species tended to have a similar foot length and claw size. However, a clustering pattern was evident at local scale for all traits, independently of the existence of a phylogenetic signal. Indeed, even small mammal species that are phylogenetically unrelated or have strict habitat preferences possess the capacity to exploit several dimensions of phenotypic space (Wilson and Sanchez-Villagra 2010). Therefore, many species with similar morphologies are able to use forest and grassland habitats opportunistically, owing to their ability to swim, dive, dig, or climb in certain circumstances (Samuels and Van Valkenburgh 2008), although adaptations for cursorial life (e.g., small foot size) tend to hamper climbing (Shapiro et al. 2014). Harsh environmental conditions and variations in vegetation height generated a clustering structure of avian communities along a beach, dune, and coastal-grassland gradient (Gianuca et al. 2013). Similarly, Graham et al. (2009) detected changes in the phylogenetic structure of hummingbird communities along an altitudinal gradient. At high altitudes, conditions such as cold climate produced a phylogenetic clustering, which resulted in assemblages composed of species that were more similar in their responses to the local vegetation structure and climatic stresses (Graham et al. 2009). The morphological and phylogenetic clustering observed here, although unexpected in the forest interior, might be an effect of free access of cattle to the grassland and forest environments. Cattle affect heterogeneity of herbaceous and understory strata through trampling and grazing, preventing the occurrence of many small mammal species (Pedó et al. 2010; Luza et al. 2014). Thus, environmental filtering leads to assemblages composed of species with phylogenetically conserved tolerances and strategies for habitat exploitation under harsh environmental conditions related to the vegetation structure in these habitats.

In the absence of environmental disturbances and harsh conditions, increases in the importance of ecological interactions (Graham et al. 2009), density-dependent, and stochastic processes (Chase 2007) are expected. However, edges do not offer environmental stability. Grassland-forest ecotones possess convoluted edges (sense Strayer et al. 2003) caused by the establishment of nurse plants and forest patches (Oliveira and Pillar 2004; Duarte et al. 2006). Forest and grassland habitats are blended near an edge, creating high heterogeneity and allowing the co-occurrence of small mammal species with different ecological requirements. Concomitantly, an edge can act as a barrier for ecological fluxes and cause changes in conditions and resources (Strayer et al. 2003), generating variations in species composition, species richness and total abundance in nonvolant small mammal assemblages across grassland-forest ecotones (Pedó et al. 2010; A. L. Luza, unpubl. data). The edges seemed to favor the co-occurrence of unrelated but morphologically similar species near the forest edge, probably determining which lineages are able to occur either in grassland or in the forest interior. Studies have found low degrees of coexistence of morphologically and phylogenetically related rodent species in habitats with low environmental heterogeneity (Kelt et al. 1995; Stevens et al. 2012). However, these contrasting patterns could arise from phytophysiognomic differences: These studies treated mainly arid environments, while we studied ecotones that are structurally quite different. In any event, ecological interactions can force the development of strategies to decrease niche overlap near the forest edge and allow species to coexist. Selection and segregation of microhabitats, allied to efficient partitioning of resources through diurnal or seasonal changes in preferred food items, may be important strategies permitting small mammals to coexist (Dalmagro and Vieira 2005; Chillo et al. 2010).

Several studies have shown that environmental filters are the predominant factors structuring communities at the landscape and region scales, due to increases in environmental heterogeneity and speciation processes, allied to dispersal limitation (Cavender-Bares et al. 2009; de Bello et al. 2013). At the landscape scale, variations in the structure of assemblages may be related to land use and changes in environmental heterogeneity (Kent et al. 2011). In our study, disturbances related to fire and/or grazing may have influenced the coexistence patterns even at landscape scale, due to the profound effects of these disturbances on habitat heterogeneity and on mosaic dynamics (Luza et al. 2014). The continuous effect of cattle grazing and trampling on the vegetation structure and the occasional but severe influence of fire events may function as environmental filters that select only species that are tolerant to postdisturbance conditions, resulting in assemblages that contain more similar species and individuals coexisting at the landscape scale than would be expected by chance. Interestingly, our results showed high morphological dissimilarity (morphological repulsion) only in the landscape where fire and cattle grazing have not occurred for at least 20 years (landscape G). In contrast, all the other landscapes, which are influenced by fire and grazing, exhibited phylogenetic and morphological clustering, indicating that disturbances are important environmental filters affecting the phylogenetic and morphological structure of small mammal assemblages at landscape scale. The repulsion observed in one landscape in Serra do Sudeste (B) and two landscapes in Campanha (C and I) should be evaluated with caution, because of the impossibility of computing confidence intervals.

In addition to the processes occurring at the landscape scale, processes acting at a broad scale, which includes topographic and climatic variations across regions, may hierarchically select species that are able to occupy local communities from an available phylogenetic and functional pool (de Bello et al. 2013). Owing to environmental filtering related to climate and increases in environmental heterogeneity, phylogenetic and functional clustering at broader scales is expected (Cavender-Bares et al. 2009). This hypothesis was supported by the morphological structure of regional assemblages, because similarities in morphologies are related to performance and ecological tolerances of organisms, which are important for regional persistence in the face of environmental restrictions (Cavender-Bares et al. 2009). Our results revealed phylogenetic niche conservatism in two regions and ecological convergence in one region (patterns of morphological clustering but phylogenetic repulsion). Niche conservatism may constrain the occupancy by certain lineages in such a way that only phylogenetically and functionally related species would co-occur in local communities, because the species' adaptations to deal with variations in environmental conditions across regions are phylogenetically conserved (Cooper et al. 2011; Davies and Buckley 2011). However, if regional the phylogenetic pool includes lineages with a high ability to colonize many regions, historical processes of colonization may place species belonging to distinct lineages (but similar morphologies) in the same region and cause a pattern of phylogenetic repulsion at broad scales (Gomez et al. 2010). Marsupial and rodent species occurred in all three regions, but in Campanha, we found clustering of morphologies across species of very distinct lineages (cricetid and dasyproctid rodents as well as didelphid opossums, particularly *D. albiventris*), which occurred in low densities but in similar frequencies. Thus, the phylogenetic repulsion found in one region (Campanha) revealed a preponderance of historical processes allowing the colonization of local assemblages in that region.

## Conclusion

Our results indicated that environmental filtering and ecological interactions were important processes structuring assemblages at the local scale. At the landscape and region scales, environmental filtering and historical processes seem to have caused variations in phylogenetic and morphological similarities among the species and individuals coexisting in these small mammal assemblages. We found clustering of morphologies across the entire gradient, and phylogenetic clustering in the grassland and forest habitats. Phylogenetically closer species tended to co-occur less than expected near the forest edge. Thus, a balance between organism's tolerances in face of environmental filtering and the decrease in niche overlap due to ecological interactions limited phylogenetic relatedness and morphological similarities between taxa at the local scale. At the landscape scale, environmental filtering seemed to be an important process structuring the small mammal assemblages. Finally, the phylogenetic and morphological structure of the small mammal assemblages revealed the influence of both environmental filtering and historical processes at the regional scale. The study also contributes to the recognition of factors that generate diversity and distribution patterns of Neotropical nonvolant small mammal assemblages.

## Data Accessibility

Amplified sequences were deposited on GenBank (accession numbers KJ936941–KJ936960). Occurrence data and individual morphological traits were deposited on DRYAD Repository (http://dx.doi.org/10.5061/dryad.nf2h5/1).

## Supporting Information

**Figure S1.** Schematic figure of the sampling grid utilized. Full circles represent the capture points.

**Figure S2.** Linear regressions showing allometric relations between morphological traits - in millimeters (log scale) against cubic root of species body mass (log scale).

**Figure S3.** Linear regressions showing allometric relations between morphological traits - in millimeters (log scale) against cubic root of individual body mass (log scale).

**Table S1.** Mean values of non-volant small mammal functional traits from grassland-forest ecotones in southern Brazil. **Table S2.** Non-volant small mammal species captured in grassland-forest ecotones in southern Brazil during spring and summer of 2011, 2012 and 2013.

**Appendix S1.** List of species, genes and the correspondent GenBank accession numbers included in the sequence dataset used to perform phylogenetic reconstruction.

**Appendix S2.** Several figures showing the structure of non-volant small-mammal assemblages at three scales depicted by each individual and species morphologic trait.

## References

[b1] Arnold SJ (1983). Morphology, performance and fitness. Am. Zool.

[b2] de Bello F, Lavorel S, Lavergne S, Albert CH, Boulangeat I, Mazel F (2013). Hierarchical effects of environmental filters on the functional structure of plant communities: a case study in the French Alps. Ecography.

[b3] Blomberg SP, Garland T, Ives AR (2003). Testing for phylogenetic signal in comparative data: behavioral traits are more labile. Evolution.

[b4] Boldrini II, Pillar VD, Müller SC, Castilhos ZMdS, Jacques AVÁ (2009). A flora dos Campos do Rio Grande do Sul. Campos Sulinos: conservação e uso sustentável da biodiversidade.

[b5] Bond WJ, Parr CL (2010). Beyond the forest edge: ecology, diversity and conservation of the grassy biomes. Biol. Conserv.

[b6] Bouckaert R, Heled J, Kühnert D, Vaughan T, Wu C-H, Xie D (2014). BEAST 2: A Software Platform for Bayesian Evolutionary Analysis. PLoS Comput. Biol.

[b7] Cavender-Bares J, Kozak KH, Fine PVA, Kembel SW (2009). The merging of community ecology and phylogenetic biology. Ecol. Lett.

[b8] Chase JM (2007). Drought mediates the importance of stochastic community assembly. Proc. Natl Acad. Sci. USA.

[b9] Chillo V, Rodriguez D, Ojeda RA (2010). Niche partitioning and coexistence between two mammalian herbivores in the Dry Chaco of Argentina. Acta Oecol.

[b10] Christoff AU, Lima Jd, Fonseca CR, Souza AF, Leal-Zanchet AM, Dutra T, Backes A, Ganade G, Jung DMH (2009). Mamíferos não-voadores da Floresta com Araucária e áreas adjacentes no Rio Grande do Sul: ênfase em roedores e suas adaptações ao habitat. Floresta com Araucária: Ecologia, Conservação e Desenvolvimento Sustentável.

[b11] Cianciaruso MV, Batalha MA, Gaston KJ, Petchey OL (2009). Including intraspecific variability in functional diversity. Ecology.

[b12] Cooper N, Rodriguez J, Purvis A (2008). A common tendency for phylogenetic overdispersion in mammalian assemblages. Proc. R. Soc. B Biol. Sci.

[b13] Cooper N, Freckleton RP, Jetz W (2011). Phylogenetic conservatism of environmental niches in mammals. Proc. R. Soc. B Biol. Sci.

[b14] Dalmagro AD, Vieira EM (2005). Patterns of habitat utilization of small rodents in an area of Araucaria forest in Southern Brazil. Austral Ecol.

[b15] Davies TJ, Buckley LB (2011). Phylogenetic diversity as a window into the evolutionary and biogeographic histories of present-day richness gradients for mammals. Philos. Trans. R. Soc. B Biol. Sci.

[b16] Davies TJ, Cooper N, Diniz-Filho JAF, Thomas GH, Meiri S (2012). Using phylogenetic trees to test for character displacement: a model and an example from a desert mammal community. Ecology.

[b17] D'Elia G (2003). Phylogenetics of sigmodontinae (Rodentia, Muroidea, Cricetidae), with special reference to the akodont group, and with additional comments on historical biogeography. Cladistics.

[b18] Diamond JM, Cody ML, Diamond JM (1975). Assembly of species communities. Ecology and Evolution of Communities.

[b19] Drummond AJ, Rambaut A (2007). BEAST: Bayesian evolutionary analysis by sampling trees. BMC Evol. Biol.

[b20] Drummond AJ, Ho SYW, Phillips MJ, Rambaut A (2006). Relaxed phylogenetics and dating with confidence. PLoS Biol.

[b21] Duarte LDS, Dos-Santos MMG, Hartz SM, Pillar VD (2006). Role of nurse plants in Araucaria Forest expansion over grassland in south Brazil. Austral Ecol.

[b22] Fabre PH, Hautier L, Dimitrov D, Douzery EJP (2012). A glimpse on the pattern of rodent diversification: a phylogenetic approach. BMC Evol. Biol.

[b23] Faith DP (1992). Conservation evaluation and phylogenetic diversity. Biol. Conserv.

[b24] Felsenstein J (1985). Confidence-limits on phylogenies – an approach using the bootstrap. Evolution.

[b25] Fritz SA, Purvis A (2010). Phylogenetic diversity does not capture body size variation at risk in the world's mammals. Proc. R. Soc. B Biol. Sci.

[b26] Gianuca AT, Dias RA, Debastiani VJ, Duarte LDS (2013). Habitat filtering influences the phylogenetic structure of avian communities across a coastal gradient in southern Brazil. Austral Ecol.

[b27] Gomez JP, Bravo GA, Brumfield RT, Tello JG, Daniel Cadena C (2010). A phylogenetic approach to disentangling the role of competition and habitat filtering in community assembly of Neotropical forest birds. J. Anim. Ecol.

[b28] Gotelli NJ (2000). Null model analysis of species co-occurrence patterns. Ecology.

[b29] Gotelli NJ, McCabe DJ (2002). Species co-occurrence: a meta-analysis of J. M. Diamond's assembly rules model. Ecology.

[b30] Graham CH, Parra JL, Rahbek C, McGuire JA (2009). Phylogenetic structure in tropical hummingbird communities. Proc. Natl Acad. Sci. USA.

[b31] Griffin PC, Griffin SC, Waroquiers C, Mills LS (2005). Mortality by moonlight: predation risk and the snowshoe hare. Behav. Ecol.

[b32] Guindon S, Dufayard J-F, Lefort V, Anisimova M, Hordijk W, Gascuel O (2010). New algorithms and methods to estimate maximum-likelihood phylogenies: assessing the performance of PhyML 3.0. Syst. Biol.

[b33] Hubbell SP (2006). Neutral theory and the evolution of ecological equivalence. Ecology.

[b34] IBGE (2002). Mapas do Clima do Brasil.

[b100] IBGE (2004). Mapas de Biomas e de.

[b35] Jansa SA, Weksler M (2004). Phylogeny of muroid rodents: relationships within and among major lineages as determined by IRBP gene sequences. Mol. Phylogenet. Evol.

[b36] Keddy PA (1992). Assembly and response rules – 2 goals for predictive community ecology. J. Veg. Sci.

[b37] Kelt DA, Taper ML, Meserve PL (1995). Assessing the impact of competition on community assembly - a case-study using small mammals. Ecology.

[b38] Kembel SW, Cowan PD, Helmus MR, Cornwell WK, Morlon H, Ackerly DD (2010). Picante: R tools for integrating phylogenies and ecology. Bioinformatics.

[b39] Kent R, Bar-Massada A, Carmel Y (2011). Multiscale analyses of mammal species composition – environment relationship in the contiguous USA. PLoS ONE.

[b40] Kraft NJB, Cornwell WK, Webb CO, Ackerly DD (2007). Trait evolution, community assembly, and the phylogenetic structure of ecological communities. Am. Nat.

[b41] Lange S, Stalleicken J, Burda H (2004). Functional morphology of the ear in fossorial rodents, *Microtus arvalis* and *Arvicola terrestris*. J. Morphol.

[b42] Leite RN, Kolokotronis S-O, Almeida FC, Werneck FP, Rogers DS, Weksler M (2014). In the wake of invasion: tracing the historical biogeography of the South American cricetid radiation (Rodentia, Sigmodontinae). PLoS ONE.

[b43] Losos JB (2008). Phylogenetic niche conservatism, phylogenetic signal and the relationship between phylogenetic relatedness and ecological similarity among species. Ecol. Lett.

[b44] Lovette IJ, Hochachka WM (2006). Simultaneous effects of phylogenetic niche conservatism and competition on avian community structure. Ecology.

[b45] Luza AL, Carlucci MB, Hartz SM, Duarte LDS (2014). Moving from forest vs. grassland perspectives to an integrated view toward the conservation of forest-grassland mosaics. Natureza & Conservação.

[b46] Mangini PR, Cullen L, Rudran R, Valladares-Padua C, Nicola PA (2003). Captura e marcação de animais silvestres. Métodos de estudos em Biologia da Conservação e Manejo da Vida Silvestre.

[b47] Mayfield MM, Levine JM (2010). Opposing effects of competitive exclusion on the phylogenetic structure of communities. Ecol. Lett.

[b48] Mitchell KJ, Pratt RC, Watson LN, Gibb GC, Llamas B, Kasper M (2014). Molecular phylogeny, biogeography, and habitat preference evolution of marsupials. Mol. Biol. Evol.

[b49] Nimer E (1979). Um modelo metodológico de classificação de climas. Rev. Bras. Geogr.

[b50] Oliveira JM, Pillar VD (2004). Vegetation dynamics on mosaics of Campos and Araucaria forest between 1974 and 1999 in Southern Brazil. Commun. Ecol.

[b51] Pavoine S, Bonsall MB (2011). Measuring biodiversity to explain community assembly: a unified approach. Biol. Rev.

[b52] Pedó E, de Freitas TRO, Hartz SM (2010). The influence of fire and livestock grazing on the assemblage of non-flying small mammals in grassland-Araucaria Forest ecotones, southern Brazil. Zoologia.

[b53] Petchey OL, Gaston KJ (2002). Functional diversity (FD), species richness and community composition. Ecol. Lett.

[b54] Pillar VD, Orloci L (1996). On randomization testing in vegetation science: multifactor comparisons of releve groups. J. Veg. Sci.

[b55] Pillar VD, Quadros FLFd (1997). Grassland-forest boundaries in southern Brazil. Coenoses.

[b56] Posada D (2008). jModelTest: phylogenetic model averaging. Mol. Biol. Evol.

[b57] Quintela FM, Goncalves GL, Althoff SL, Sbalqueiro IJ, Oliveira LFB, de Freitas TRO (2014). A new species of swamp rat of the genus Scapteromys Waterhouse, 1837 (Rodentia: Sigmodontinae) endemic to *Araucaria angustifolia* Forest in Southern Brazil. Zootaxa.

[b58] R Development Core Team (2011). R: A language and environment for statistical computing.

[b59] Rambaut A (2009). http://tree.bio.ed.ac.uk/software/figtree.

[b60] Samuels JX, Van Valkenburgh B (2008). Skeletal indicators of locomotor adaptations in living and extinct rodents. J. Morphol.

[b61] Shapiro LJ, Young JW, VandeBerg JL (2014). Body size and the small branch niche: using marsupial ontogeny to model primate locomotor evolution. J. Hum. Evol.

[b62] Steppan SJ, Adkins RM, Anderson J (2004). Phylogeny and divergence-date estimates of rapid radiations in muroid rodents based on multiple nuclear genes. Syst. Biol.

[b63] Stevens RD, Gavilanez MM, Tello JS, Ray DA (2012). Phylogenetic structure illuminates the mechanistic role of environmental heterogeneity in community organization. J. Anim. Ecol.

[b64] Strayer DL, Power ME, Fagan WF, Pickett STA, Belnap J (2003). Bioscience.

[b65] Szalay FS (1994). Evolutionary history of the marsupials and an analysis of osteological characters.

[b66] Taraborelli P, Corbalan V, Giannoni S (2003). Locomotion and escape modes in rodents of the Monte desert (Argentina). Ethology.

[b67] Violle C, Enquist BJ, McGill BJ, Jiang L, Albert CH, Hulshof C (2012). The return of the variance: intraspecific variability in community ecology. Trends Ecol. Evol.

[b68] Webb CO, Ackerly DD, McPeek MA, Donoghue MJ (2002). Phylogenies and community ecology. Annu. Rev. Ecol. Syst.

[b69] Weksler M (2003). Phylogeny of Neotropical oryzomyine rodents (Muridae: Sigmodontinae) based on the nuclear IRBP exon. Mol. Phylogenet. Evol.

[b70] Wilson LAB, Sanchez-Villagra MR (2010). Diversity trends and their ontogenetic basis: an exploration of allometric disparity in rodents. Proc. R. Soc. B Biol. Sci.

