# Supplementary figures and images for: Phylogenetic and morphological relationships between nonvolant small mammals reveal assembly processes at different spatial scales
Source: Ecol Evol. 2015 Jan 25;5(4):889–902. doi: 10.1002/ece3.1407 (PMC4338971; doi:10.1002/ece3.1407)

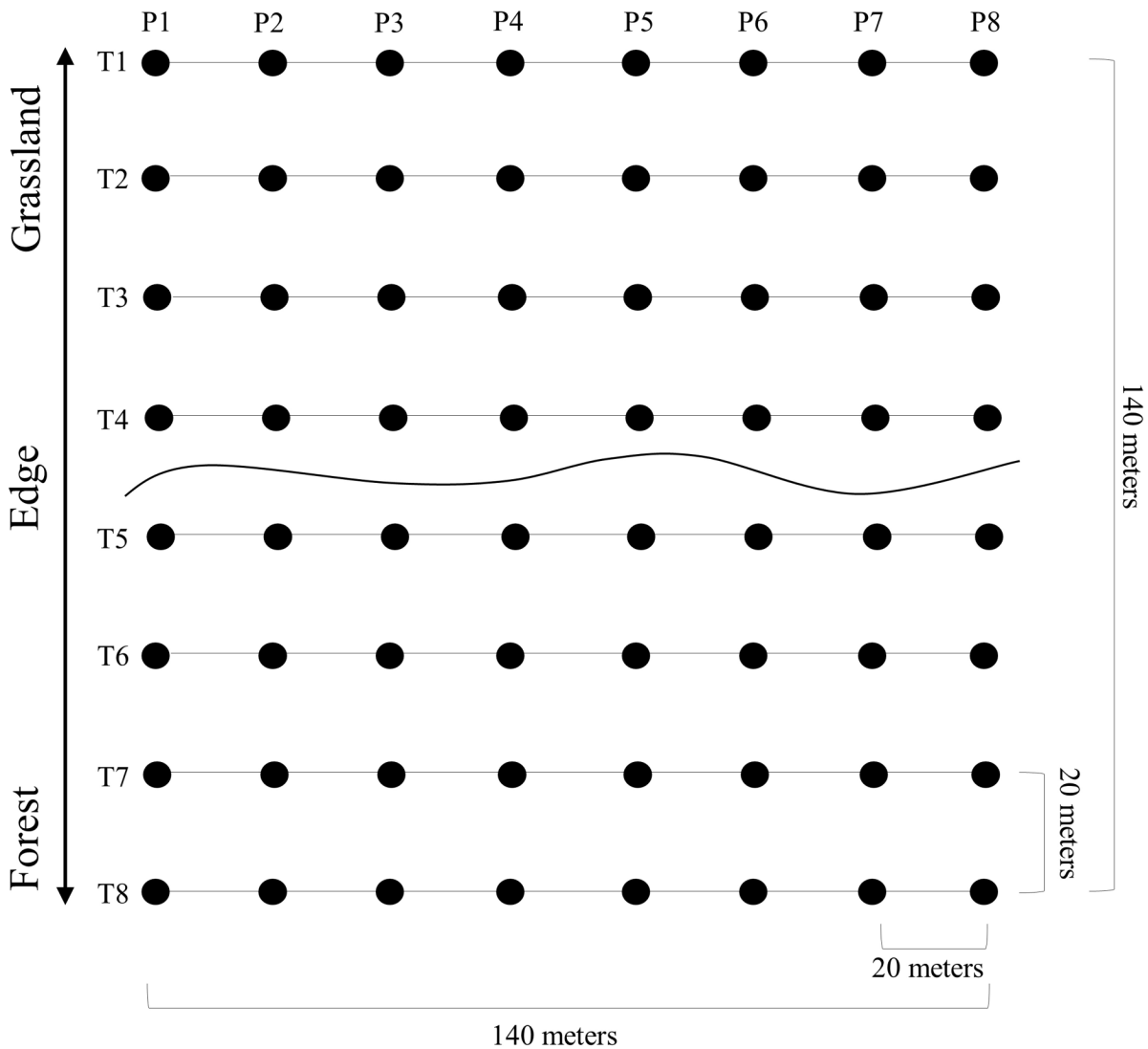

Supplement: Supplementary file 1 [file ece30005-0889-sd1.pdf]

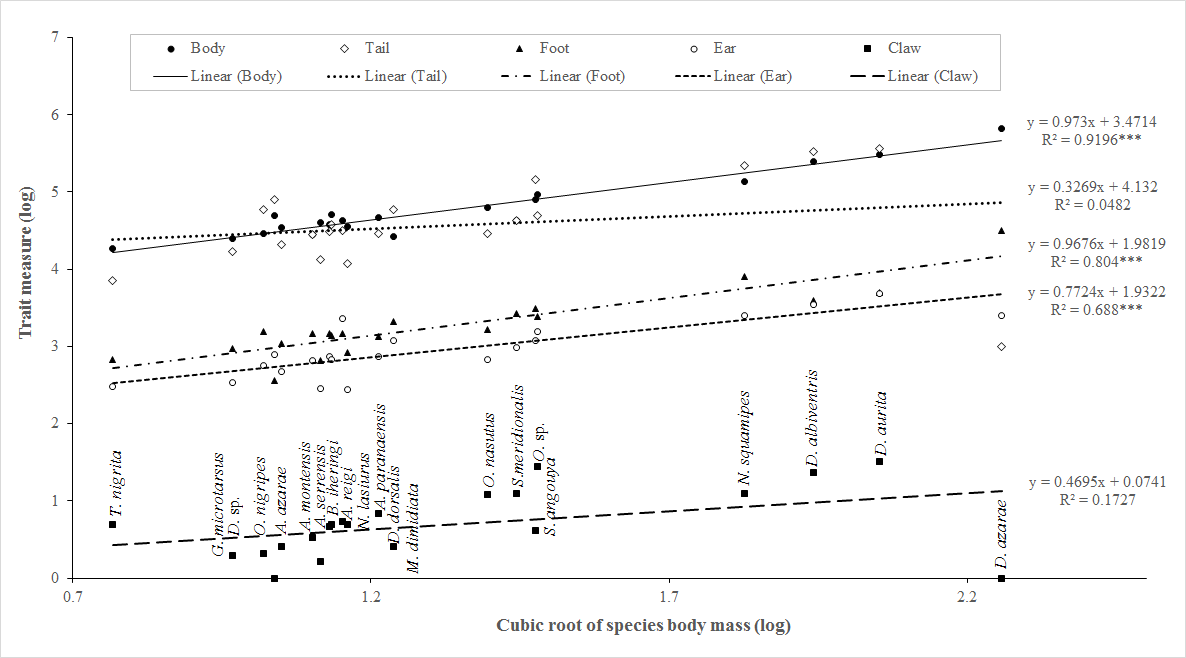

Supplement: Supplementary file 2 [file ece30005-0889-sd2.tif]

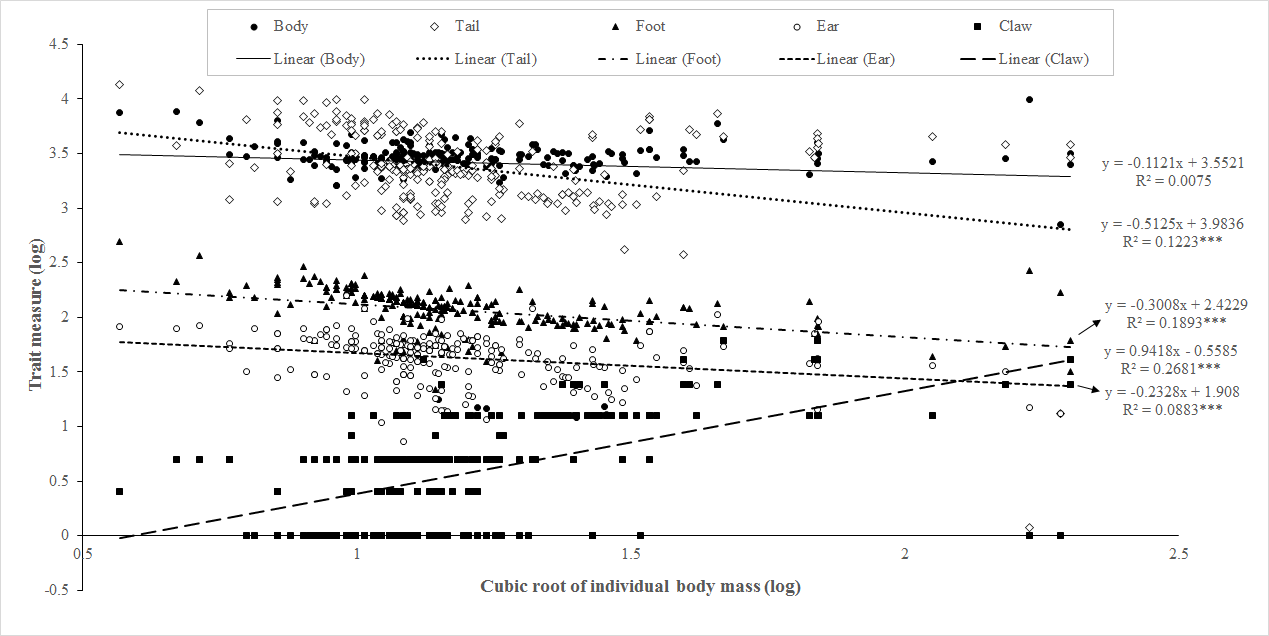

Supplement: Supplementary file 3 [file ece30005-0889-sd3.tif]
