# Supplementary material for: Phylogenetic and morphological relationships between nonvolant small mammals reveal assembly processes at different spatial scales
Source: Ecol Evol. 2015 Jan 25;5(4):889–902. doi: 10.1002/ece3.1407 (PMC4338971; doi:10.1002/ece3.1407)
Supplement: Supplementary file 4 [file ece30005-0889-sd4.docx]

**Tables**

**Table S1**: Mean values of non-volant small-mammal functional traits from grassland-forest ecotones in southern Brazil.

| Family/species | | Functional traits | | | | | |
| --- | --- | --- | --- | --- | --- | --- | --- |
| Didelphidae | | Tail length (mm) | Body length (mm) | Posterior foot length (mm) | Claw size (mm) | Ear size (mm) | Body mass (g.) |
|  | *Didelphis albiventris* (Lund 1840) | 250.83 | 220.00 | 36.36 | 3.92 | 34.50 | 338.92 |
|  | *Didelphis aurita* (Wied-Neuwied 1826) | 260.00 | 240.00 | 40.50 | 4.50 | 40.00 | 473.00 |
|  | *Gracilinanus microtarsus* (Wagner 1872) | 135.00 | 110.00 | 13.00 | 1.00 | 18.00 | 22.50 |
|  | *Monodelphis dimidiata* (Wagner 1847) | 61.74 | 99.70 | 16.76 | 1.24 | 11.69 | 28.37 |
| Cricetidae | |  |  |  |  |  |  |
|  | *Akodon azarae* (Fischer 1829) | 75.50 | 93.20 | 21.00 | 1.50 | 14.50 | 23.35 |
|  | *Akodon montensis* (Thomas 1913) | 89.33 | 97.75 | 23.86 | 1.96 | 17.54 | 29.79 |
|  | *Akodon paranaensis* (Christoff *et al*. 2000) | 86.31 | 106.63 | 22.81 | 2.31 | 17.55 | 38.06 |
|  | *Akodon reigi*  (González, Langguth and Oliveira 1998) | 90.08 | 103.15 | 23.85 | 2.08 | 29.00 | 31.73 |
|  | *Akodon serrensis* (Thomas 1902) | 86.00 | 85.73 | 23.77 | 1.68 | 16.73 | 27.27 |
|  | *Brucepattersonius iheringi* (Thomas 1897) | 97.00 | 110.50 | 23.00 | 2.00 | 17.00 | 30.00 |
|  | *Delomys dorsalis* (Hensel 1872) | 119.00 | 83.43 | 27.71 | 1.50 | 21.57 | 40.93 |
|  | *Deltamys* sp. (Thomas 1917) | 68.67 | 80.83 | 19.67 | 1.33 | 12.58 | 18.25 |
|  | *Necromys lasiurus* (Lund 1841) | 59.00 | 95.50 | 18.50 | 2.00 | 11.50 | 32.50 |
|  | *Nectomys squamipes* (Brantz 1872) | 210.00 | 170.00 | 50.00 | 3.00 | 30.00 | 240.00 |
|  | *Oligoryzomys nigripes* (Olfers 1818) | 118.60 | 86.96 | 24.28 | 1.38 | 15.76 | 21.28 |
|  | *Oxymycterus nasutus* (Waterhouse 1837) | 86.15 | 120.96 | 25.17 | 2.94 | 17.00 | 65.92 |
|  | *Oxymycterus* sp. (Waterhouse 1837) | 109.00 | 143.75 | 29.50 | 4.25 | 24.25 | 84.50 |
|  | *Scapteromys meridionalis* (Quintela *et al*. 2014) | 103.00 | 102.25 | 30.75 | 3.00 | 19.75 | 76.25 |
|  | *Sooretamys angouya* (Thomas 1913) | 173.71 | 134.57 | 32.86 | 1.86 | 21.71 | 83.86 |
|  | *Thaptomys nigrita* (Lichtenstein 1829) | 47.00 | 71.00 | 17.00 | 2.00 | 12.00 | 10.00 |
| Dasyproctidade | |  |  |  |  |  |  |
|  | *Dasyprocta azarae* (Lichtenstein 1823) | 20.00 | 337.00 | 90.00 | 1.00 | 30.00 | 875.00 |

**Table S2**: Non-volant small-mammal species captured in grassland-forest ecotones in southern Brazil during spring and summer of 2011, 2012 and 2013. § = Mean capture success (%).

|  | Region | A | | B | | C | | A | | B | | C | | A | | B | | C | | Presence sum |
| --- | --- | --- | --- | --- | --- | --- | --- | --- | --- | --- | --- | --- | --- | --- | --- | --- | --- | --- | --- | --- |
|  | Landscape | A | | B | | C | | D | | E | | F | | G | | H | | I | |  |
|  | Sampling grid | G1 | G2 | G1 | G2 | G1 | G2 | G1 | G2 | G1 | G2 | G1 | G2 | G1 | G2 | G1 | G2 | G1 | G2 |  |
| Family/Species | |  |  |  |  |  |  |  |  |  |  |  |  |  |  |  |  |  |  |  |
| Didelphidae | |  |  |  |  |  |  |  |  |  |  |  |  |  |  |  |  |  |  |  |
|  | *Didelphis albiventris* (Lund 1840) |  |  |  | 1 |  |  | 1 | 1 | 1 | 1 |  |  |  |  |  |  |  | 1 | 6 |
|  | *Didelphis aurita*  (Wied-Neuwied 1826) |  |  |  |  |  |  |  | 1 |  |  |  |  |  |  |  |  |  |  | 1 |
|  | *Gracilinanus microtarsus* (Wagner 1872) |  | 1 |  |  |  |  |  |  |  |  |  |  |  |  |  |  |  |  | 1 |
|  | *Monodelphis dimidiata* (Wagner 1847) |  | 1 |  |  |  |  |  |  |  |  |  |  | 1 | 1 |  |  |  |  | 3 |
| Cricetidae | |  |  |  |  |  |  |  |  |  |  |  |  |  |  |  |  |  |  |  |
|  | *Akodon azarae*  (Fischer 1829) | 1 | 1 |  |  |  |  |  |  |  |  |  |  | 1 | 1 |  |  |  |  | 4 |
|  | *Akodon montensis*  (Thomas 1913) |  |  |  |  | 1 |  |  |  |  |  |  |  | 1 | 1 |  |  | 1 | 1 | 5 |
|  | *Akodon paranaensis* (Christoff *et al*. 2000) | 1 |  |  |  |  |  | 1 | 1 |  |  |  |  |  |  |  |  |  |  | 3 |
|  | *Akodon reigi*  (González, Langguth and Oliveira 1998) |  |  |  | 1 |  |  |  |  | 1 |  |  |  |  |  |  | 1 |  |  | 3 |
|  | *Akodon serrensis*  (Thomas 1902) |  |  |  |  |  |  |  |  |  |  |  |  | 1 | 1 |  |  |  |  | 2 |
|  | *Akodon* sp.  (Meyen 1833) |  |  |  |  |  |  |  |  |  |  |  |  | 1 | 1 |  |  |  |  | 2 |
|  | *Brucepattersonius iheringi* (Thomas 1897) | 1 |  |  |  |  |  |  |  |  |  |  |  |  |  |  |  |  |  | 1 |
|  | *Delomys dorsalis*  (Hensel 1872) |  |  |  |  |  |  | 1 | 1 |  |  |  |  | 1 | 1 |  |  |  |  | 4 |
|  | *Deltamys* sp.  (Thomas 1917) |  | 1 |  |  |  |  |  |  |  |  |  |  | 1 | 1 |  |  |  |  | 3 |
|  | *Necromys lasiurus*  (Lund 1841) |  |  |  |  |  |  |  | 1 |  |  |  |  |  |  |  |  |  |  | 1 |
|  | *Nectomys squamipes* (Brantz 1872) |  |  |  |  | 1 |  |  |  |  |  |  |  |  |  |  |  |  |  | 1 |
|  | *Oligoryzomys nigripes* (Olfers 1818) | 1 |  |  | 1 | 1 |  | 1 | 1 | 1 |  |  |  | 1 | 1 | 1 | 1 |  |  | 10 |
|  | *Oxymycterus nasutus* (Waterhouse 1837) | 1 | 1 | 1 |  |  |  |  |  |  |  |  |  | 1 | 1 |  |  |  |  | 5 |
|  | *Oxymycterus* sp. (Waterhouse 1837) |  |  |  |  |  |  |  | 1 |  |  |  |  |  |  |  |  |  |  | 1 |
|  | *Scapteromys meridionalis* (Quintela *et al.* 2014) |  |  |  |  |  |  | 1 |  |  |  |  |  | 1 | 1 |  |  |  |  | 3 |
|  | *Sooretamys angouya* (Thomas 1913) |  |  |  |  |  |  | 1 | 1 | 1 |  |  |  |  |  |  | 1 |  |  | 4 |
|  | *Thaptomys nigrita* (Lichtenstein 1823) |  |  |  |  |  |  |  |  |  |  |  |  | 1 |  |  |  |  |  | 1 |
| Dasyproctidae | |  |  |  |  |  |  |  |  |  |  |  |  |  |  |  |  |  |  |  |
|  | *Dasyprocta azarae* (Lichtenstein 1823) |  |  |  |  | 1 |  |  |  |  | 1 |  |  |  |  |  |  |  |  | 2 |
| Species numbers | | 5 | 5 | 1 | 3 | 4 | 0 | 6 | 8 | 4 | 2 | 0 | 0 | 11 | 10 | 1 | 3 | 1 | 2 | 22 |
| Individual numbers | | 8 | 9 | 1 | 7 | 5 | 0 | 26 | 25 | 11 | 2 | 0 | 0 | 73 | 40 | 1 | 21 | 1 | 6 | 236 |
| Capture numbers | | 8 | 12 | 2 | 7 | 6 | 0 | 35 | 28 | 12 | 5 | 0 | 0 | 93 | 60 | 1 | 27 | 1 | 9 | 306 |
| Capture success (%) | | 0.66 | 0.73 | 0.09 | 0.62 | 0.44 | 0 | 2.04 | 2.1 | 0.98 | 0.18 | 0 | 0 | 8.42 | 3.98 | 0.1 | 2.3 | 0.08 | 0.6 | 1.3 § |
