# Supplementary material for: Phylogenetic and morphological relationships between nonvolant small mammals reveal assembly processes at different spatial scales
Source: Ecol Evol. 2015 Jan 25;5(4):889–902. doi: 10.1002/ece3.1407 (PMC4338971; doi:10.1002/ece3.1407)
Supplement: Supplementary file 5 [file ece30005-0889-sd5.docx]

Appendix S1

List of species, genes and the correspondent GenBank acession numbers included in the sequence dataset used to perform phylogenetic reconstruction.

|  |  |  | **Genbank acession number** | | |
| --- | --- | --- | --- | --- | --- |
|  |  |  | **Gene** | | |
| **Order** | **Genus** | **Species** | **Cytb** | **COI** | **IRBP** |
| Rodentia |  |  |  |  |  |
|  | *Akodon* | *azarae* | AY702964 | GU938990 | KJ614590 |
|  |  | *montensis* | EU251018 | GU938934 | KF815409 |
|  |  | *paranaensis* | EU579471 | GU938993 | EU649035 |
|  |  | *reigi* | AY195865 | - | - |
|  |  | *serrensis* | EF101889 | KF815397 | KF815411 |
|  | *Brucepattersonius* | *iheringi* | KJ936943 | - | - |
|  | *Dasyprocta* | *azarae* | KJ936945 | - | - |
|  | *Delomys* | *dorsalis* | KF317031 | GU938952 | KF815411 |
|  | *Deltamys* | sp. | KJ936955 | - | - |
|  | *Necromys* | *lasiurus* | KF815437 | GU938997 | KF815415 |
|  | *Nectomys* | *squamipes* | EU074634 | JF491612 | KF815416 |
|  | *Oligoryzomys* | *nigripes* | KF815443 | KF815406 | KF815419 |
|  | *Oxymycterus* | *nasutus* | EF661854 | - | AY277468 |
|  |  | sp. | KJ936948 | - | - |
|  | *Scapteromys* | *meridionalis* | KJ936957 | - | - |
|  | *Sooretamys* | *angouya* | KF815444 | GU938962 | KF815420 |
|  | *Thaptomys* | *nigrita* | EF206815 | KF815403 | KF815421 |
| Didelphimorphia |  |  |  |  |  |
|  | *Didelphis* | *albiventris* | KM071410 | JN638922 | AF257683 |
|  |  | *aurita* | GU112881 | GU112798 | - |
|  | *Gracilinanus* | *microtarsus* | KF313982 | GU112804 | AF257687 |
|  | *Monodelphis* | *dimidiata* | KM071564 | - | KM071077 |
