# Supplementary material for: Phylogenetic and morphological relationships between nonvolant small mammals reveal assembly processes at different spatial scales
Source: Ecol Evol. 2015 Jan 25;5(4):889–902. doi: 10.1002/ece3.1407 (PMC4338971; doi:10.1002/ece3.1407)
Supplement: Supplementary file 6 [file ece30005-0889-sd6.docx]

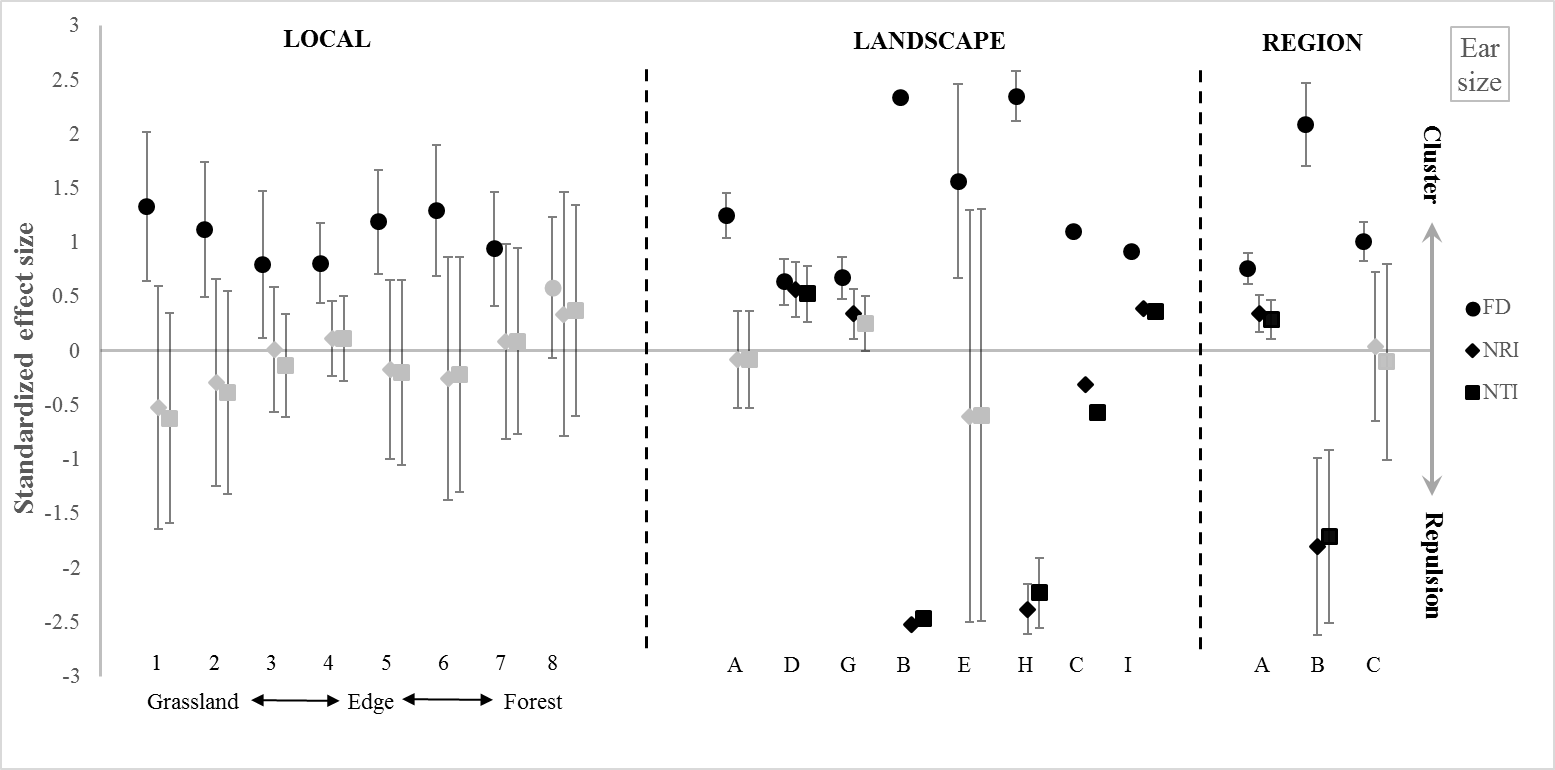


Morphologic structure of non-volant small-mammal assemblages at three scales based on species ear size. Ear size showed no phylogenetic pattern. Bars are equivalent to confidence intervals of 95 %. Black symbols indicate index values different from the random expectation. FD = Species Functional Diversity; NRI = Species Net Relatedness Index; NTI = Species Nearest Taxon Index.


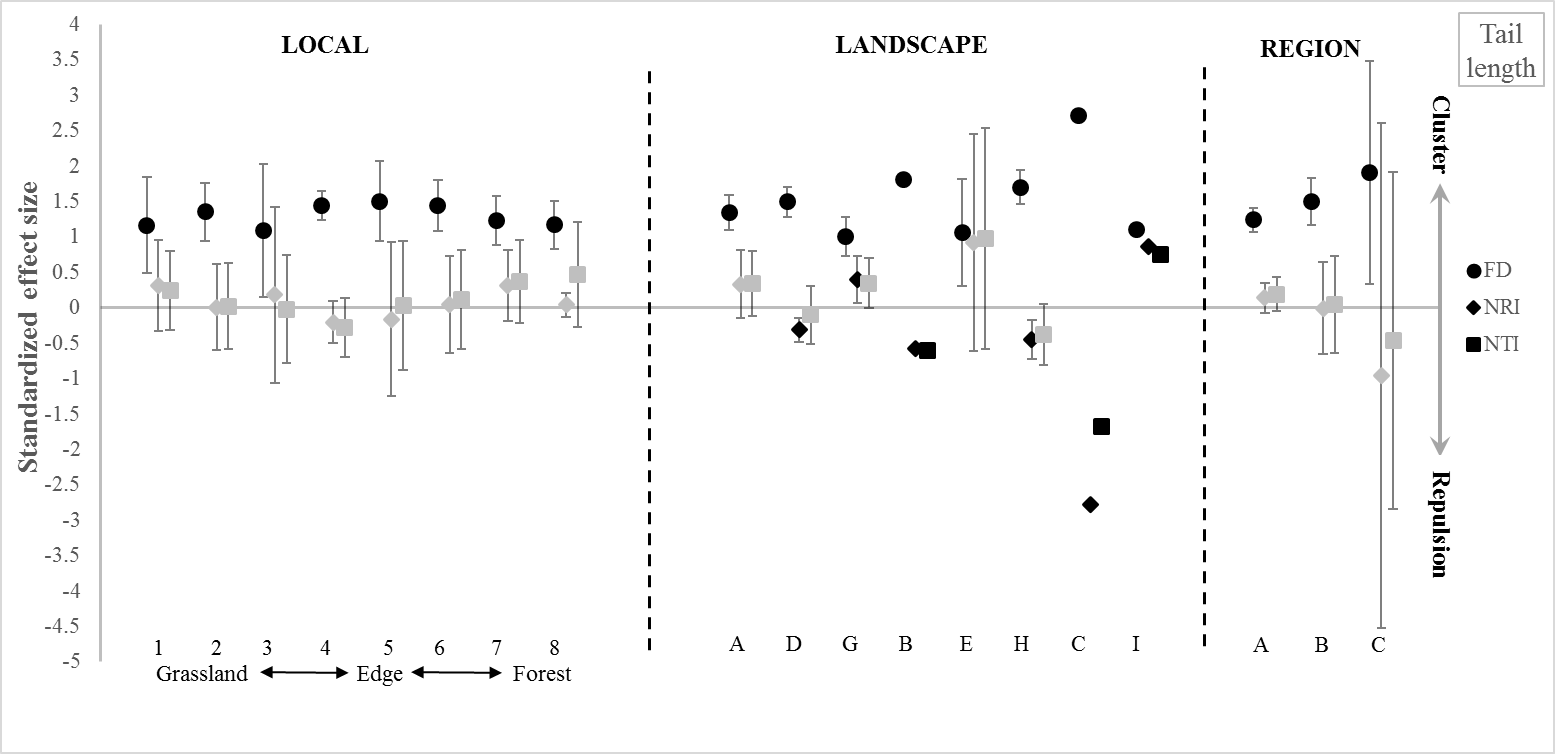


Morphologic structure of non-volant small-mammal assemblages at three scales based on species tail length. Tail length was less conserved than expected by Brownian motion model. Bars are equivalent to confidence intervals of 95 %. Black symbols indicate index values different from the random expectation. FD = Species Functional Diversity; NRI = Species Net Relatedness Index; NTI = Species Nearest Taxon Index.


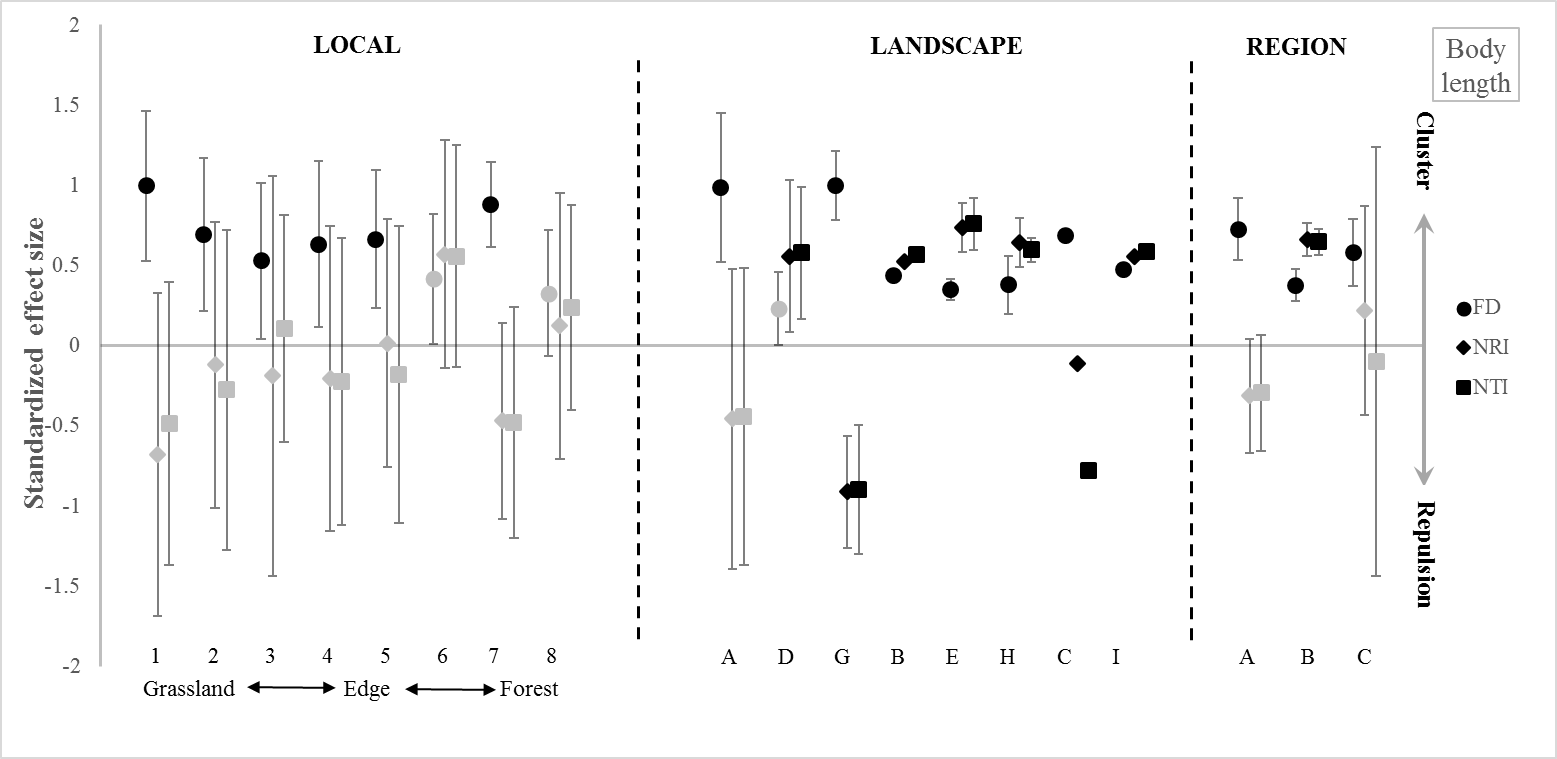


Morphologic structure of non-volant small-mammal assemblages at three scales based on species body length. Body length was less conserved than expected by Brownian motion model. Bars are equivalent to confidence intervals of 95 %. Black symbols indicate index values different from the random expectation. FD = Species Functional Diversity; NRI = Species Net Relatedness Index; NTI = Species Nearest Taxon Index.


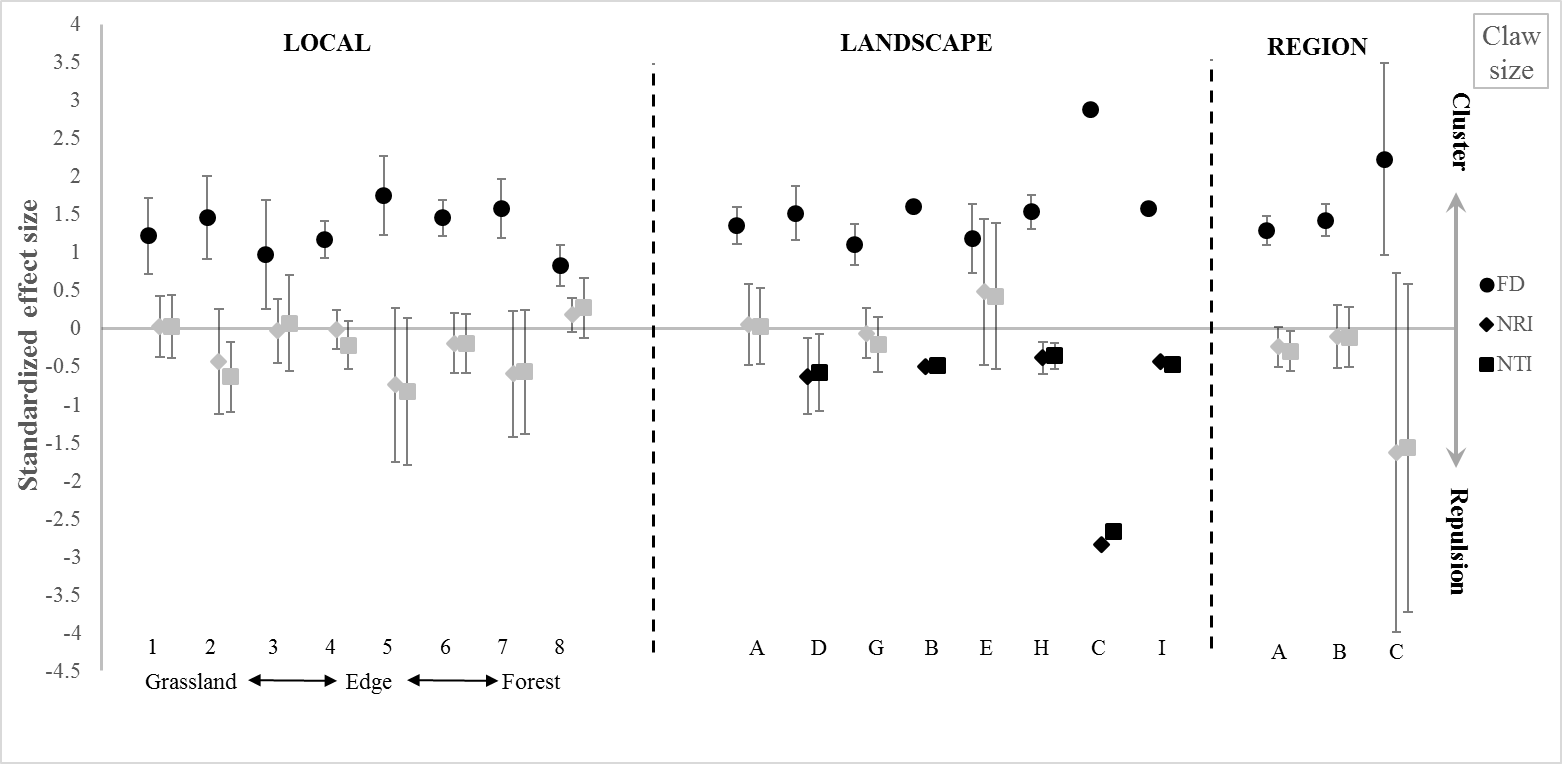


Morphologic structure of non-volant small-mammal assemblages at three scales based on species claw size. Claw size was more conserved than expected by Brownian motion model. Bars are equivalent to confidence intervals of 95 %. Black symbols indicate index values different from the random expectation. FD = Species Functional Diversity; NRI = Species Net Relatedness Index; NTI = Species Nearest Taxon Index.


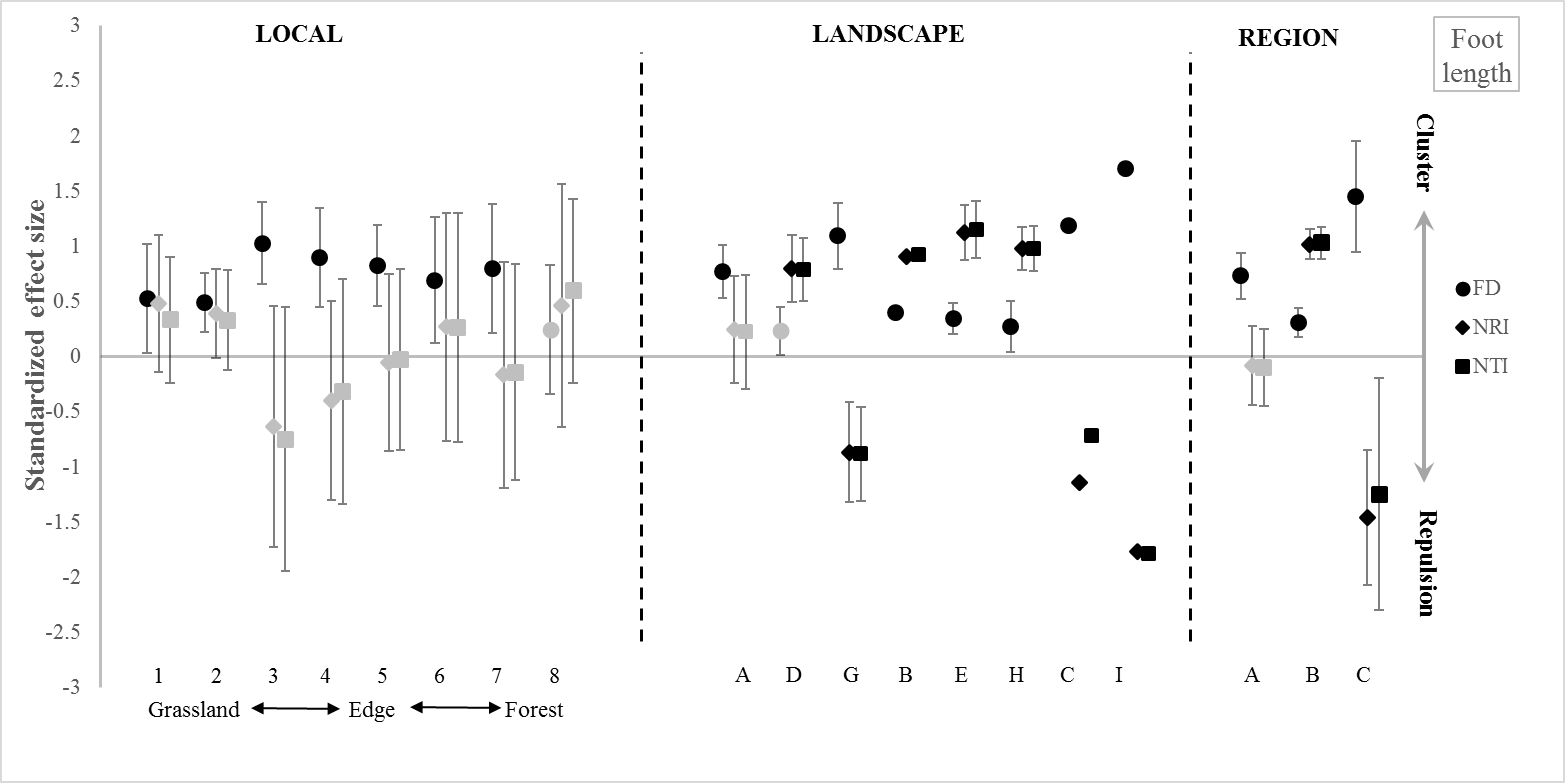


Morphologic structure of non-volant small-mammal assemblages at three scales based on species foot length. Foot length was more conserved than expected by Brownian motion model. Bars are equivalent to confidence intervals of 95 %. Black symbols indicate index values different from the random expectation. FD = Species Functional Diversity; NRI = Species Net Relatedness Index; NTI = Species Nearest Taxon Index.


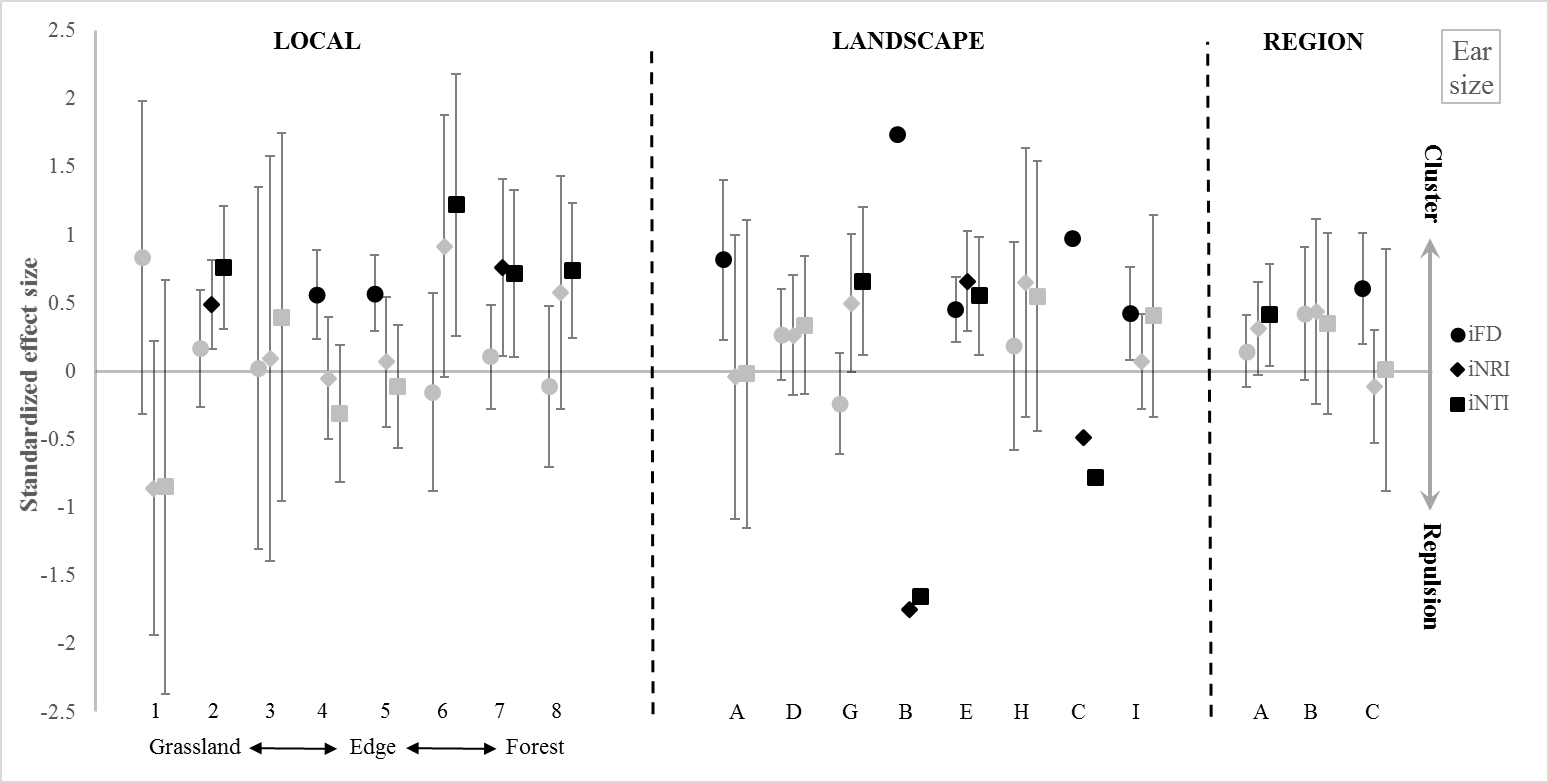


Morphologic structure of non-volant small-mammal assemblages at three scales based on individual ear size. Bars are equivalent to confidence intervals of 95 %. Black symbols indicate index values different from the random expectation. iFD = Individuals Functional Diversity; iNRI = Individual Net Relatedness Index; iNTI = Individual Nearest Taxon Index.


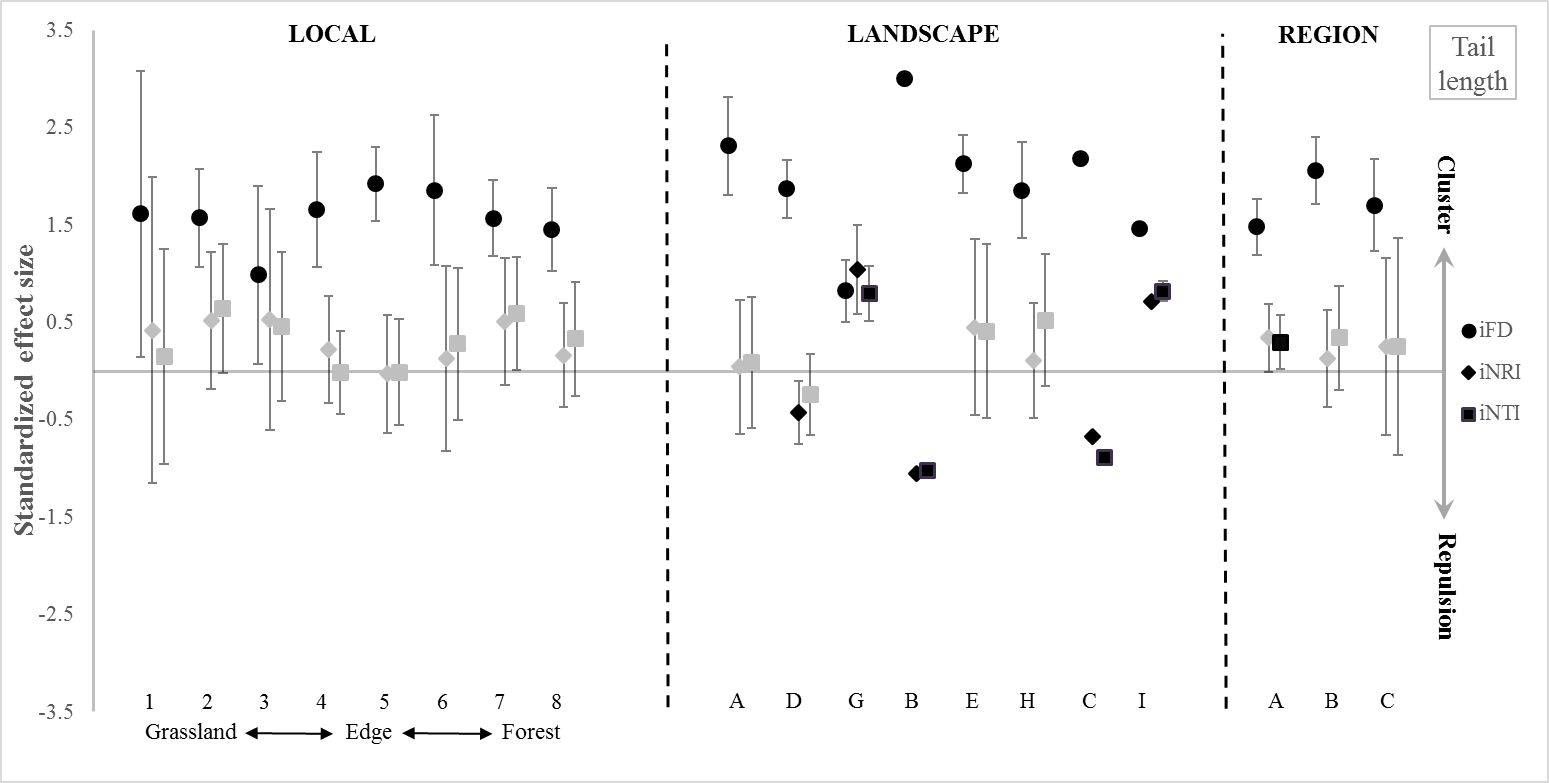


Morphologic structure of non-volant small-mammal assemblages at three scales based on individual tail length. Bars are equivalent to confidence intervals of 95 %. Black symbols indicate index values different from the random expectation. iFD = Individuals Functional Diversity; iNRI = Individual Net Relatedness Index; iNTI = Individual Nearest Taxon Index.


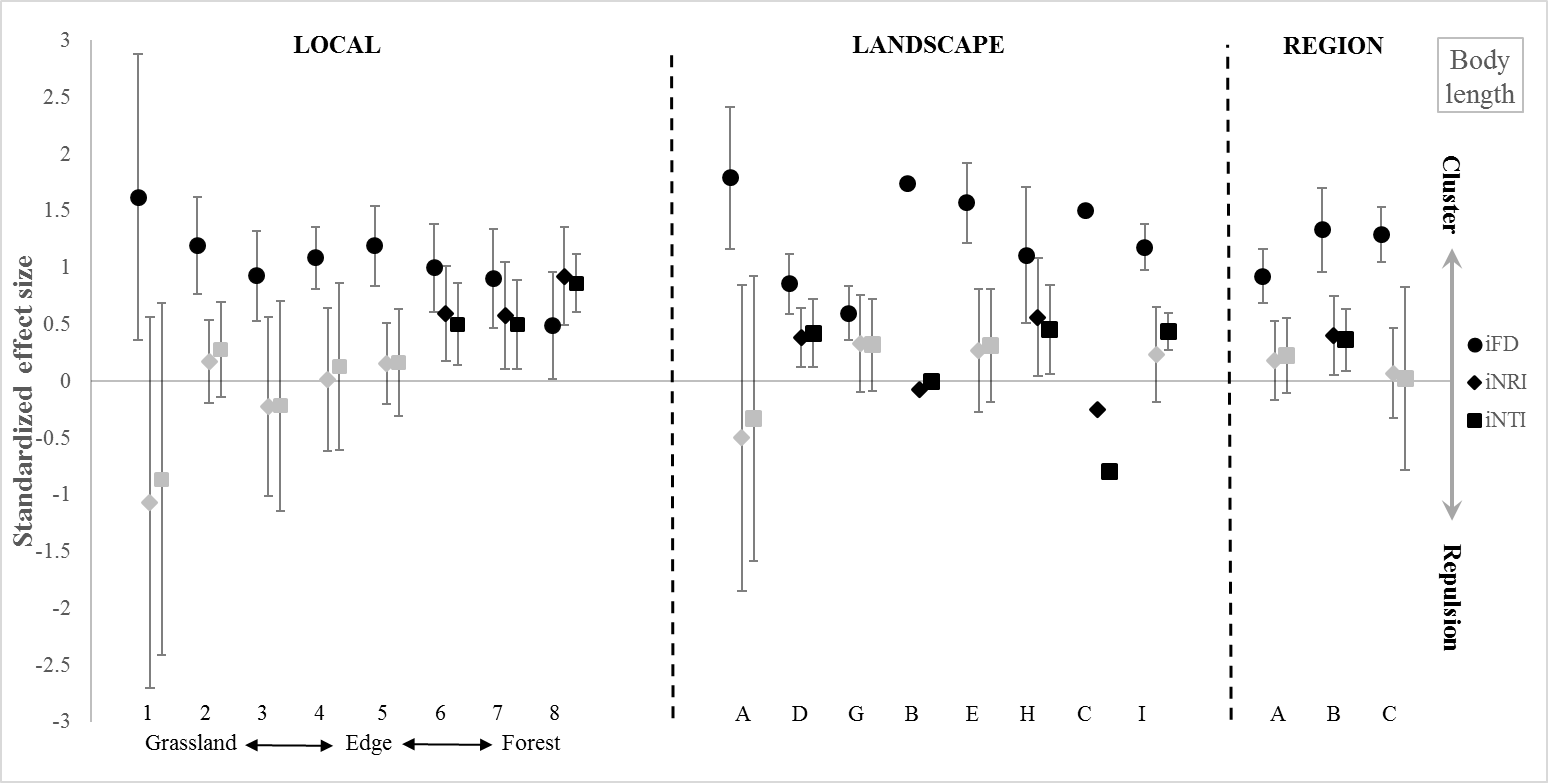


Morphologic structure of non-volant small-mammal assemblages at three scales based on individual ear size. Bars are equivalent to confidence intervals of 95 %. Black symbols indicate index values different from the random expectation. iFD = Individuals Functional Diversity; iNRI = Individual Net Relatedness Index; iNTI = Individual Nearest Taxon Index.


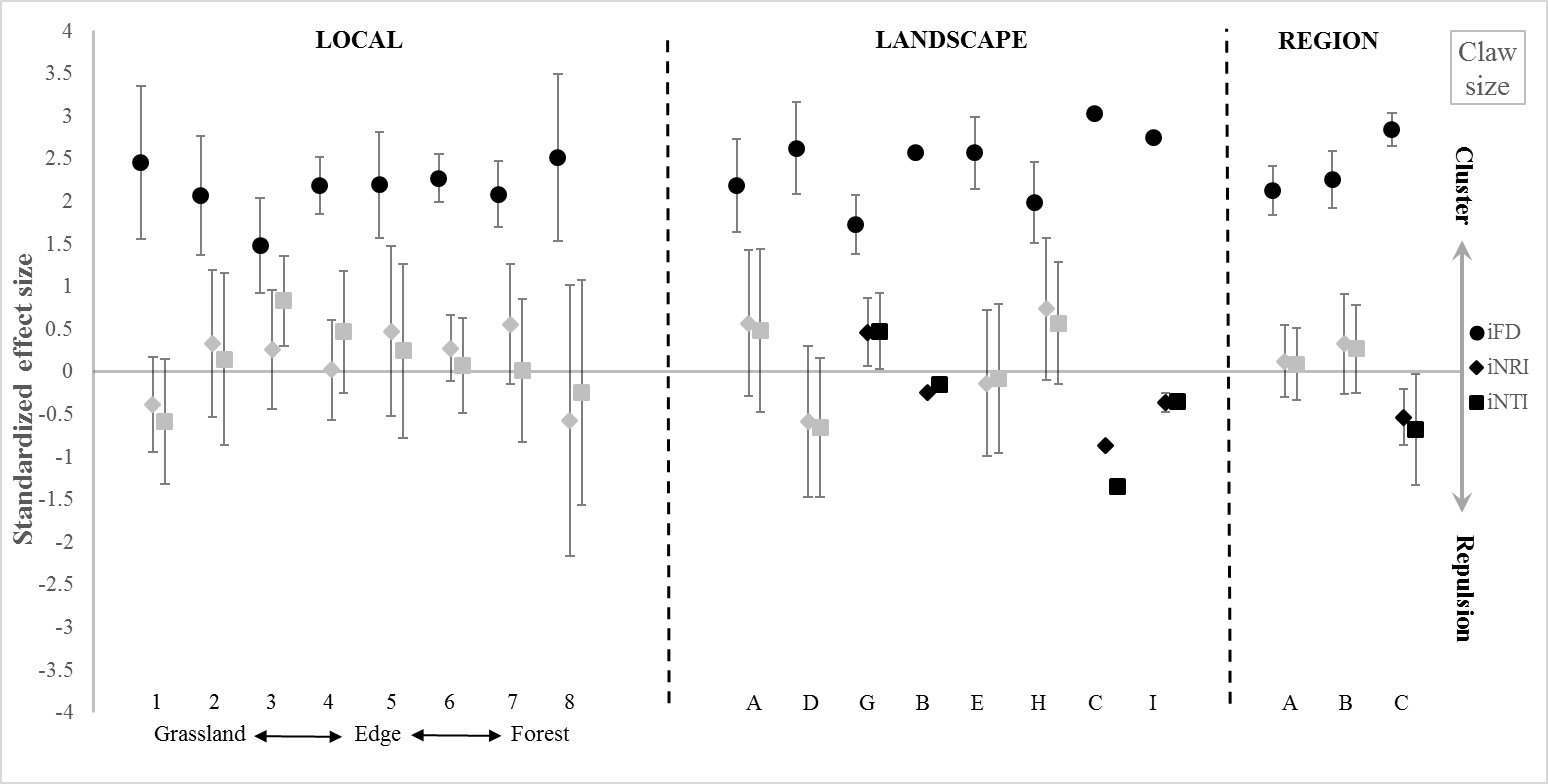


Morphologic structure of non-volant small-mammal assemblages at three scales based on individual claw size. Bars are equivalent to confidence intervals of 95 %. Black symbols indicate index values different from the random expectation. iFD = Individuals Functional Diversity; iNRI = Individual Net Relatedness Index; iNTI = Individual Nearest Taxon Index.


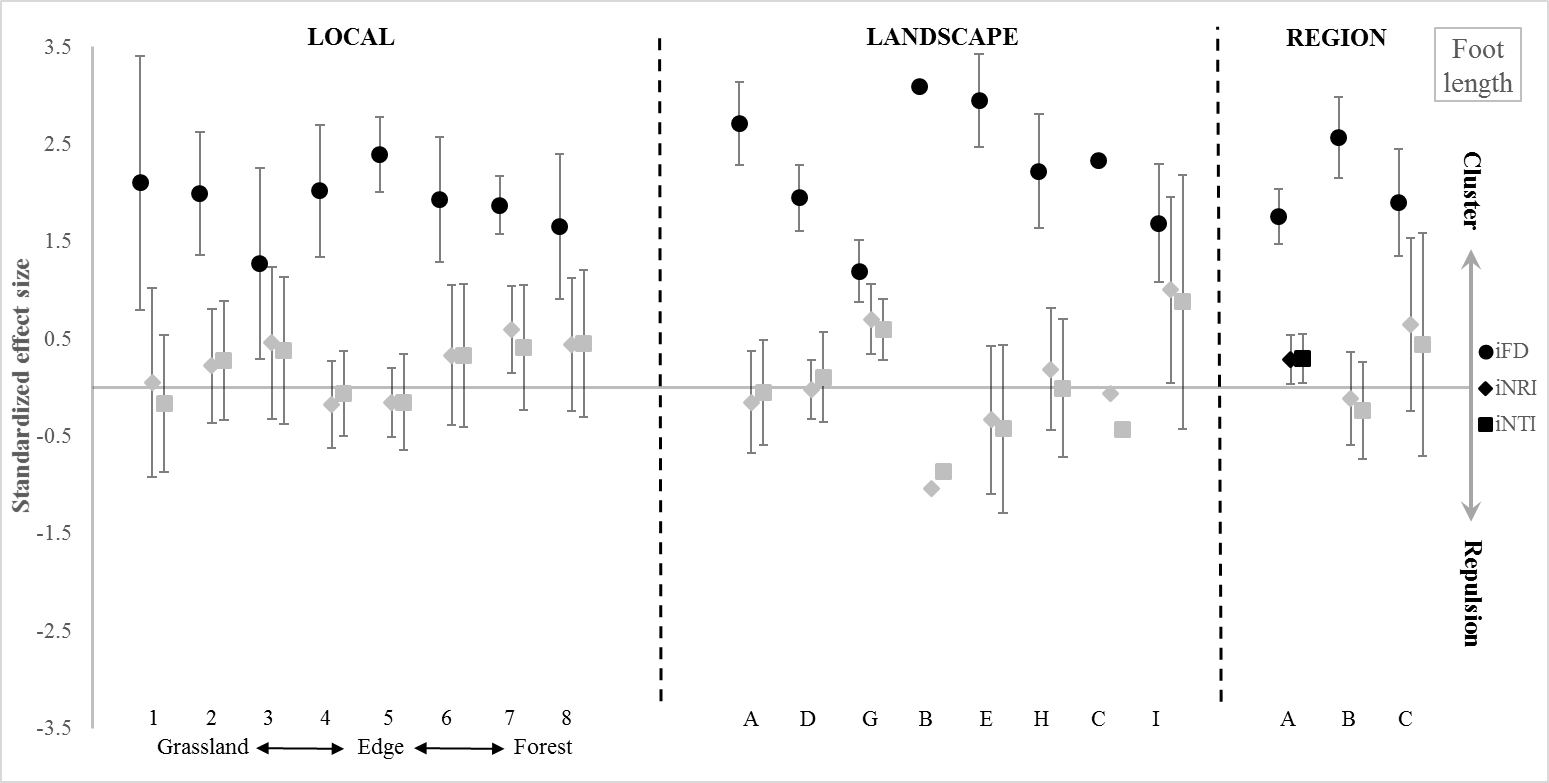


Morphologic structure of non-volant small-mammal assemblages at three scales based on individual foot length. Bars are equivalent to confidence intervals of 95 %. Black symbols indicate index values different from the random expectation. iFD = Individuals Functional Diversity; iNRI = Individual Net Relatedness Index; iNTI = Individual Nearest Taxon Index.
